# Innovative Business Idea Generation in Higher Education: The Interplay between Entrepreneurship Centres and Economic Conditions

**DOI:** 10.12688/f1000research.167767.1

**Published:** 2025-09-03

**Authors:** Jane Ogochukwu. Ben-Caleb, Itumeleng Ngowi, Adebanji A.W. Ayeni, Ayandeji Sunday Ayantokun, Inegbedion Henry E., Egbide Ben-Caleb

**Affiliations:** 1 Business Administration, Landmark University College of Business and Social Sciences, Omu-Aran, Kwara, Nigeria; 2 Business Support Studies, Central University of Technology Free State Faculty of Management Sciences, Bloemfontein, Free State, South Africa; 3 Business School, North West University Faculty of Economic and Management Sciences, Potchefstroom, North West, South Africa; 4 Business Administration, Wigwe University, Isiokpo, Rivers State, Nigeria; 5 Business Administration, Bowen University, Iwo, Osun, Nigeria; 6 Accounting and Finance, Landmark University College of Business and Social Sciences, Omu-Aran, Kwara, Nigeria

**Keywords:** Youth Entrepreneurship, Entrepreneurship Centres, Economic Conditions, Higher Education, Social Innovation, Nigeria

## Abstract

**Background:**

Understanding how entrepreneurship centres in developing economies shape students’ entrepreneurial capabilities, especially under difficult economic conditions, remains a key concern in higher education research. This study examines the role of entrepreneurship centres in Nigerian universities in fostering innovative business idea generation among undergraduates. Guided by Ecological Systems Theory, entrepreneurship centres are viewed as part of students’ immediate (microsystem) environment, while broader economic challenges such as inflation, insecurity, power instability, and high interest rates form the exosystem context.

**Methods:**

A cross-sectional design was adopted, with data collected from 269 final-year students across selected Nigerian universities using a structured questionnaire. Partial Least Squares Structural Equation Modelling (PLS-SEM) was applied to assess both direct effects and the moderating influence of economic conditions.

**Results:**

The results show that active engagement with entrepreneurship centres significantly boosts students’ ability to generate innovative business ideas. However, adverse economic conditions not only influence idea generation on their own but also weaken the positive impact of entreprenuership centre engagement. The model demonstrated moderate explanatory power (R ^2^ = 0.416) and strong predictive relevance (Q ^2^ = 0.466), supported by robust reliability and validity metrics.

**Conclusions:**

This study highlights the need for universities and policymakers to build more resilient entrepreneurship support systems capable of nurturing innovation despite economic headwinds. The findings point to the value of sustained investment in entrepreneurship centres and call for broader research across different contexts to enhance generalisability.

## 1. Introduction

Entrepreneurship education (EE) has increasingly become a strategic mechanism for promoting innovation, self-reliance, and youth employment across developing economies, particularly in Nigeria. ^1^ In response to persistent graduate unemployment and the saturation of the formal labour market, the Nigerian National Universities Commission (NUC) mandated the integration of entrepreneurship education into all tertiary curricula beginning in the 2007/2008 academic session. ^2, 3^ Despite widespread adoption of this policy, outcomes have remained suboptimal, ^4^ estimates that approximately 33% of graduates remain unemployed, suggesting a disconnect between the policy objectives and the practical realities of graduate enterprise development. ^5, 6^

To operationalise the EE agenda and enhance its efficacy, Nigerian universities have established entrepreneurship centres (ECs), also referred to as innovation hubs or idea laboratories, intended to offer experiential learning, mentorship, access to digital infrastructure, and startup incubation support. ^7– 9^ These centres are designed to cultivate entrepreneurial competencies and promote ideation that addresses real-world problems through innovation. However, the performance of ECs cannot be assessed in isolation; their impact is shaped by the broader entrepreneurial ecosystem. Structural economic challenges such as inflation, power supply instability, insecurity, and fluctuating interest rates represent formidable constraints that may either suppress or stimulate entrepreneurial behaviour depending on the resilience of students and institutional capacity. ^10– 12^ Theoretically, entrepreneurship centres function as institutional catalysts located within the microsystem of the educational environment. However, their effectiveness is modulated by distal contextual variables that lie within the exosystem, namely, macroeconomic and structural conditions. Overlooking these contextual factors may lead to an inflated perception of the effectiveness of entrepreneurship education interventions. Prior research underscores this concern, highlighting that although tens of thousands of Nigerian students participated in EE between 2006 and 2010, the rate of startup formation remained minimal, largely due to insufficient stimulation of innovative thinking and limited ecosystemic support. ^13^

Recent studies call for a paradigmatic re-evaluation of EE structures in Nigeria, particularly those institutional components such as ECs that hold potential to drive engagement, creativity, and entrepreneurial agency. ^14, 15^ Despite some recognition of their value, the complex interdependence between ECs and contextual constraints remains underexplored in Nigerian higher education research.

Against this backdrop, this study examines how engagement with entrepreneurship centres influences innovative business idea generation among university students, with a specific focus on the moderating role of general economic conditions (GEC). Anchored in Bronfenbrenner’s ecological systems theory (EST), the study conceptualises entrepreneurship centres as proximal institutional environments (microsystems) and general economic conditions as distal structural influences (exosystems), offering a multi-layered lens through which to understand entrepreneurial development in higher education.

## 2. Literature review and hypotheses development

### 2.1 Entrepreneurship centres as learning ecosystems

Entrepreneurship centres (ECs) are increasingly embedded in higher education systems as institutional mechanisms to foster entrepreneurial mindsets and facilitate the transition from ideation to venture creation. ^16^ These centres provide experiential learning platforms where students engage in practical activities such as design thinking, opportunity recognition, mentorship programmes, and business incubation. Within the Nigerian context, ECs are a direct response to national directives, particularly the NUC’s entrepreneurship education mandate, aimed at equipping graduates with market-responsive skills. ^17^

Empirical studies highlight a positive association between student engagement in EC activities and entrepreneurial intention. ^18, 19^ However, institutional barriers such as infrastructural deficiencies, inadequate funding, and uneven access continue to challenge the scalability and impact of ECs on students’ entrepreneurial initiatives. ^20, 21^ In light of these, this study proposes the following hypothesis:

H _1_: Engagement with entrepreneurship centres significantly and positively influences innovative business idea generation among university students.

### 2.2 Innovative business idea generation

Innovative business idea generation refers to the capacity of individuals to conceptualise original, context-sensitive, and feasible business ventures. This process is both cognitive and environmental, relying on creativity, problem-solving, and access to supportive ecosystems. ^22, 23^ Research suggests that students exposed to dynamic and resource-rich entrepreneurial environments demonstrate higher levels of ideation and entrepreneurial activity. ^24^ As such, entrepreneurship centres act as enabling ecosystems where idea generation is cultivated through immersion, feedback, and risk-taking.

### 2.3 General economic conditions as a moderating variable

General economic conditions (GECs), within the EST framework, are situated in the exosystem encompassing external forces that indirectly influence the individual by shaping institutional contexts and behavioural norms. ^25^ In Nigeria, these include macroeconomic stressors such as high inflation, volatile interest rates, erratic power supply, and chronic insecurity. ^26^

Although ECs offer students resources and learning environments conducive to entrepreneurship, the broader economic landscape can either enable or inhibit their application. For example, inflationary pressures may deter startup investments, while unstable electricity may hinder operational capacity. Conversely, GEC may also induce necessity-driven entrepreneurship, whereby students innovate out of compulsion rather than opportunity. ^27^

The dual nature of GEC suggests a moderating influence on the efficacy of institutional interventions like ECs. ^28^ Drawing on this understanding, two hypotheses were advanced:

H _2_: General economic conditions significantly influence innovative business idea generation among students.

H _3_ **:**
General economic conditions significantly moderate the relationship between entrepreneurship centre engagement and innovative business idea generation.

## 3. Theoretical framework: Ecological Systems Theory (EST)

This study adopts Bronfenbrenner’s (1994) Ecological Systems Theory (EST) to conceptualize the multilevel interaction between institutional supports and contextual constraints in shaping entrepreneurial outcomes. ^25^ EST posits that human development occurs within nested systems (microsystem, mesosystem, exosystem, and macrosystem) that interact dynamically. In this framework, entrepreneurship centres are conceptualised as microsystem-level interventions, providing direct engagement, mentorship, and ideation infrastructure to students. Conversely, general economic conditions function within the exosystem, indirectly shaping students’ entrepreneurial capacity through macroeconomic realities such as resource availability, inflation, and regulatory stability. ^29, 30^ The macrosystem encompasses national educational and economic policy orientations, such as the NUC entrepreneurship education directive, which frames institutional priorities and societal expectations. ^31^

By embedding EC engagement and GEC within this theoretical structure, the study offers a comprehensive model to investigate how institutional interventions interact with structural constraints to influence innovative business idea generation in higher education.

**Figure 1.**
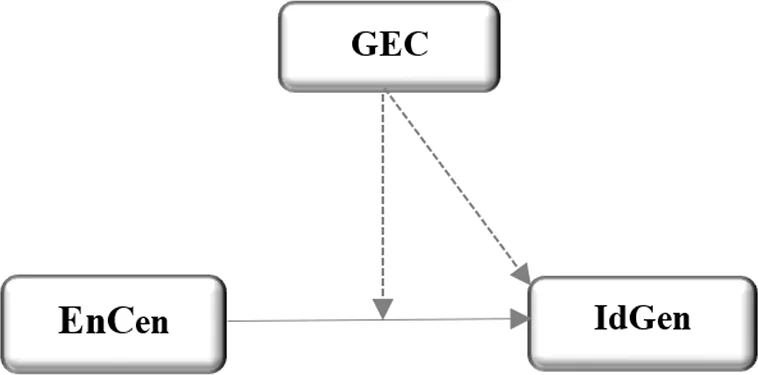
Conceptual framework. N.B: EnCen = Entrepreneurship centre; IdGen = Innovative business idea generation. This figure shows the conceptual model that outlines the hypothesised relationships examined in the study.

Figure 1 shows the conceptual model that outlines the hypothesised relationships examined in the study. It proposes that engagement with entrepreneurship centres (EnCen) has a direct positive effect on innovative business idea generation (IdGen) among university students. The model incorporates general economic conditions (GEC), captured through indicators such as inflation, insecurity, power instability, and high interest rates as a moderating variable. Specifically, it is hypothesised that GEC moderate the relationship between entrepreneurship centre engagement and innovative business idea generation.

## 4. Methodology

### 4.1 Research design and paradigm

This study adopted a quantitative, cross-sectional research design to investigate the influence of Entrepreneurship Centre Engagement (EnCen) on Innovative Business Idea Generation (IdGen) among students in Nigerian higher education institutions, with general economic conditions (GEC) assessed as a potential moderating variable. The research is anchored in the positivist paradigm, emphasising objective measurement, hypothesis testing, and the generalizability of findings across similar educational contexts in emerging economies. This paradigm supports the validation of theoretical propositions through empirical data and statistical inference.

### 4.2 Sampling procedure and data collection

Data were collected between February and March 2025 from undergraduate students enrolled in Business Administration, Accounting, and Economics programmes at a university within Nigeria’s North-Central geopolitical zone. These disciplines were purposively selected based on prior empirical evidence indicating a high propensity for entrepreneurial ideation among students in business-related fields. ^23, 32^

A total of 269 valid responses were obtained from students who had been exposed to both entrepreneurship education and institutional entrepreneurial infrastructure, such as entrepreneurship centres. The sample comprised 105 males (39.03%) and 164 females (60.97%), all aged between 20 and 25 years, reflecting the typical age range for undergraduate students in Nigerian universities. The demographic breakdown is presented in Table 1.

**Table 1.** Sample characteristics.

Variable	Frequency	Percentage (%)
**Gender**		
Male	105	39.03%
Female	164	60.97%
**Age**		
< 20 years	0	0%
20-25 years	269	100%
> 25 years	0	0%
**Department**		
Business Administration	108	40.15%
Accounting	98	36.43%
Economics	63	23.42%

A purposive sampling technique was employed to target students with exposure to entrepreneurship development studies (EDS). Participation was voluntary and anonymous, with informed consent obtained prior to questionnaire administration. Given the minimal risk involved and the observational nature of the study, formal institutional ethical clearance was not mandated under prevailing university guidelines.

**Ethical approval**

This study received ethical clearance from the Landmark University Institutional Research Ethics Committee (LMUIREC) under Ref. No: LMUIREC/HS/031/2025, dated 09–06–2025. The protocol was reviewed and approved under the oversight of the Landmark University Centre for Research Innovations and Discoveries (LUCRID). All data collection procedures adhered to the university’s ethical guidelines, and informed consent was obtained from all participants.

### 4.3 Instruments and measures

Data were collected via a structured, self-administered questionnaire adapted from previously validated scales and modified for contextual relevance. It measured three latent constructs where EnCen is the independent variable, IdGen (dependent variable) and GEC (moderating variable).

**Entrepreneurship Centre Engagement (EnCen):**

Captured students’ exposure to training, access to digital tools, and frequency of interaction with the centre. ^8, 18^ A sample statement is “I regularly participate in training sessions offered by the entrepreneurship centre”.

**Innovative Business Idea Generation (IdGen)**:

Assessed creative thinking, confidence in idea development, and novelty frequency. ^33, 34^ A sample statement is “I frequently come up with new business ideas”.

**General Economic Conditions (GEC):**

Measured students’ perceptions of macroeconomic factors such as inflation, insecurity, power instability, and interest rates. ^26, 35, 36^ A sample statement is “Rising inflation and the cost of living make it difficult to finance a business idea”.

All items were rated using a five-point Likert scale (1 = Strongly Disagree to 5 = Strongly Agree). A pilot study involving 25 students was conducted to assess clarity, internal consistency, and contextual appropriateness, leading to minor refinements. Details of the scale items are presented in Appendix A.

### 4.4 Data analysis procedure

Data were analysed using Partial Least Squares Structural Equation Modeling (PLS-SEM) in SmartPLS 4.0. ^37^ This method was selected for its suitability in handling small-to-moderate sample sizes, tolerance for non-normal data, and its robustness in modeling complex relationships including moderation. ^38, 39^ Moreover, PLS-SEM is appropriate for exploratory theory development and prediction when using reflective measurement models.

Model evaluation proceeded in two stages, as recommended by Ref. 39. In the first stage, the measurement model was evaluated to ensure the reliability and validity of the reflective constructs. Internal consistency was assessed using Cronbach’s alpha and Composite Reliability (CR), both of which confirmed acceptable levels of reliability. Convergent validity was examined through the Average Variance Extracted (AVE), ensuring that each construct explained a substantial portion of the variance in its indicators. To establish discriminant validity, the Heterotrait-Monotrait (HTMT) ratio was employed, following the threshold guidelines proposed by Ref. 40, and results confirmed adequate distinction between the constructs.

In the second stage, the structural model was assessed to test the hypothesised relationships. This involved a bootstrapping procedure with 5,000 resamples to estimate the path coefficients, effect sizes (f ^2^), and statistical significance of each relationship. The model’s explanatory power was then evaluated using the coefficient of determination (R ^2^) to determine the proportion of variance explained in the dependent variable, and predictive relevance (Q ^2^) was assessed to confirm the model’s capability in forecasting outcomes.

The study utilised eighteen indicators across the three constructs, with all statistical thresholds meeting acceptable benchmarks for reliability and validity.

To enhance the predictive robustness of the structural model, the PLSpredict algorithm was applied to generate out-of-sample predictive performance. The model’s Root Mean Square Error (RMSE) and Mean Absolute Error (MAE) were benchmarked against linear regression and mean-based models. Results confirmed the model’s adequate predictive capability for the endogenous construct. ^41, 42^

## 5. Results

### 5.1 Preliminary data diagnostics

**Figure 2.**
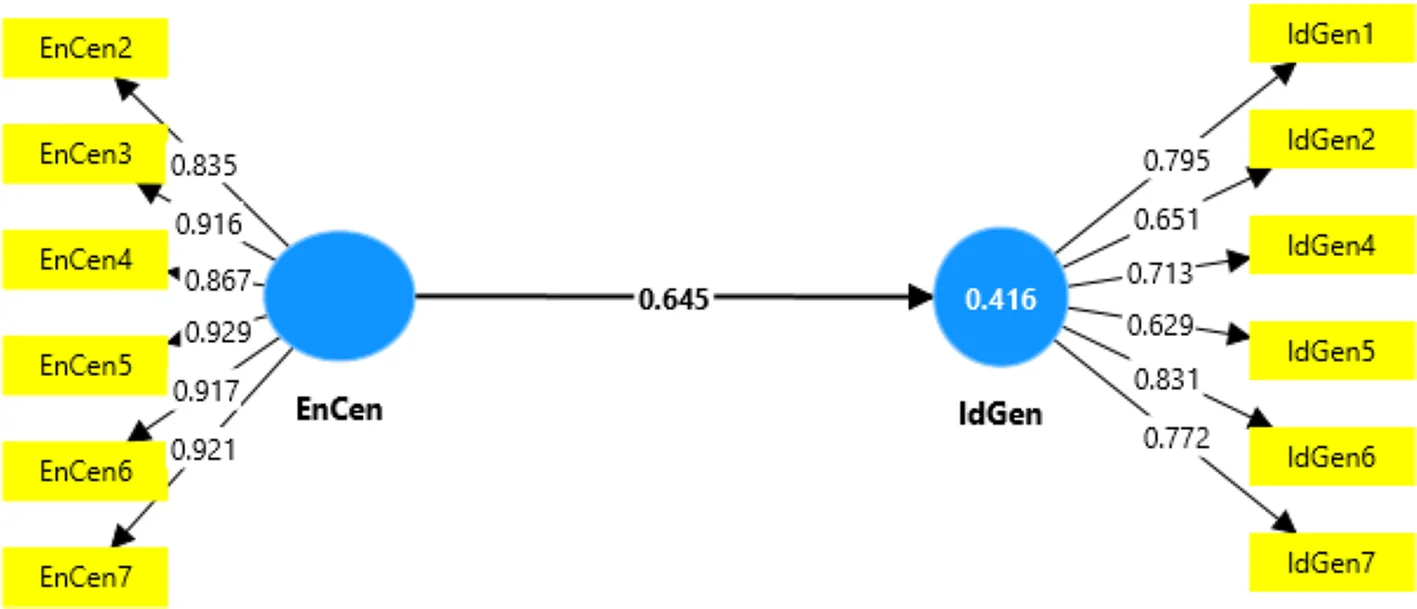
Measurement model showing indicator loadings and R ^2^ values. This figure presents the final measurement model after excluding EnCen1 and IdGen3 due to low factor loadings (< 0.60). Indicator loadings and R ^2^ values are shown to reflect the model’s construct reliability and explanatory power.

The final dataset consisted of 269 valid responses with no missing values. Descriptive analysis confirmed that the data distribution met acceptable thresholds for PLS-SEM analysis. Two measurement indicators, EnCen1 (loading = 0.188) and IdGen3 (loading = 0.596), were excluded from the analysis due to factor loadings below the acceptable threshold of 0.60. These adjustments, along with the final indicator loadings and R ^2^ values, are presented in Figure 2. In addition, the removal decision was primarily to improve the model’s quality, as recommended by Refs. 39 and 41.

### 5.2 Measurement model evaluation

The measurement model was assessed to determine the reliability and validity of the constructs using standard PLS-SEM criteria. These assessments are presented in Table 2.

**Table 2.** Reliability and validity outcome.

Variable	Cronbach’s alpha (α)	Rho_a	Rho_c	AVE	HTMT
EnCen	0.952	0.954	0.961	0.806	
IdGen	0.836	0.876	0.875	0.541	0.664
**Threshold**	**>0.70**	**>0.70**	**>0.70**	**>0.50**	**<0.85**

Internal consistency reliability was evaluated through Cronbach‘s alpha (α) and composite reliability (ρc), as recommended by. ^41^ While Cronbach’s alpha has known limitations, the use of ρc helps address underestimation concerns. Both EnCen and IdGen demonstrated high reliability, with α values of 0.952 and 0.836 respectively, and ρc values of 0.961 and 0.875, exceeding the recommended threshold of 0.70, indicating acceptable reliability.

Convergent validity was confirmed through the Average Variance Extracted (AVE), which exceeded the 0.50 benchmark for both constructs. EnCen recorded an AVE of 0.806, while IdGen had 0.541, indicating that the indicators accounted for more than half of the variance in their respective constructs, confirming convergent validity. Discriminant validity was further assessed using the Heterotrait-Monotrait (HTMT) ratio, which is considered a more robust approach than traditional methods such as the Fornell-Larcker criterion and cross-loadings. The HTMT value of 0.664 fell well below the conservative threshold of 0.85, suggesting no discriminant validity issues, in line with the criteria established by. ^41, 43^

### 5.3 Structural model evaluation

**Figure 3.**
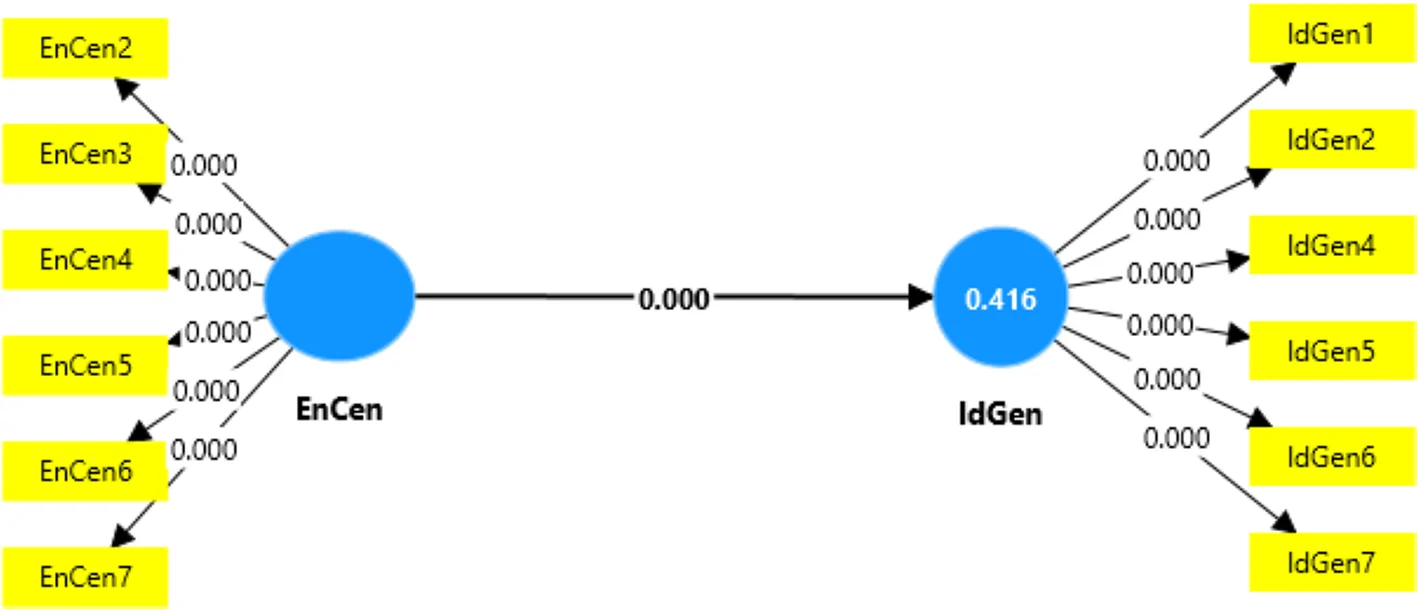
Structural model evaluating direct effects. This figure illustrates the direct relationship between entrepreneurship centre engagement and innovative business idea generation, using PLS-SEM with 5,000 bootstrap resamples.

**Figure 4.**
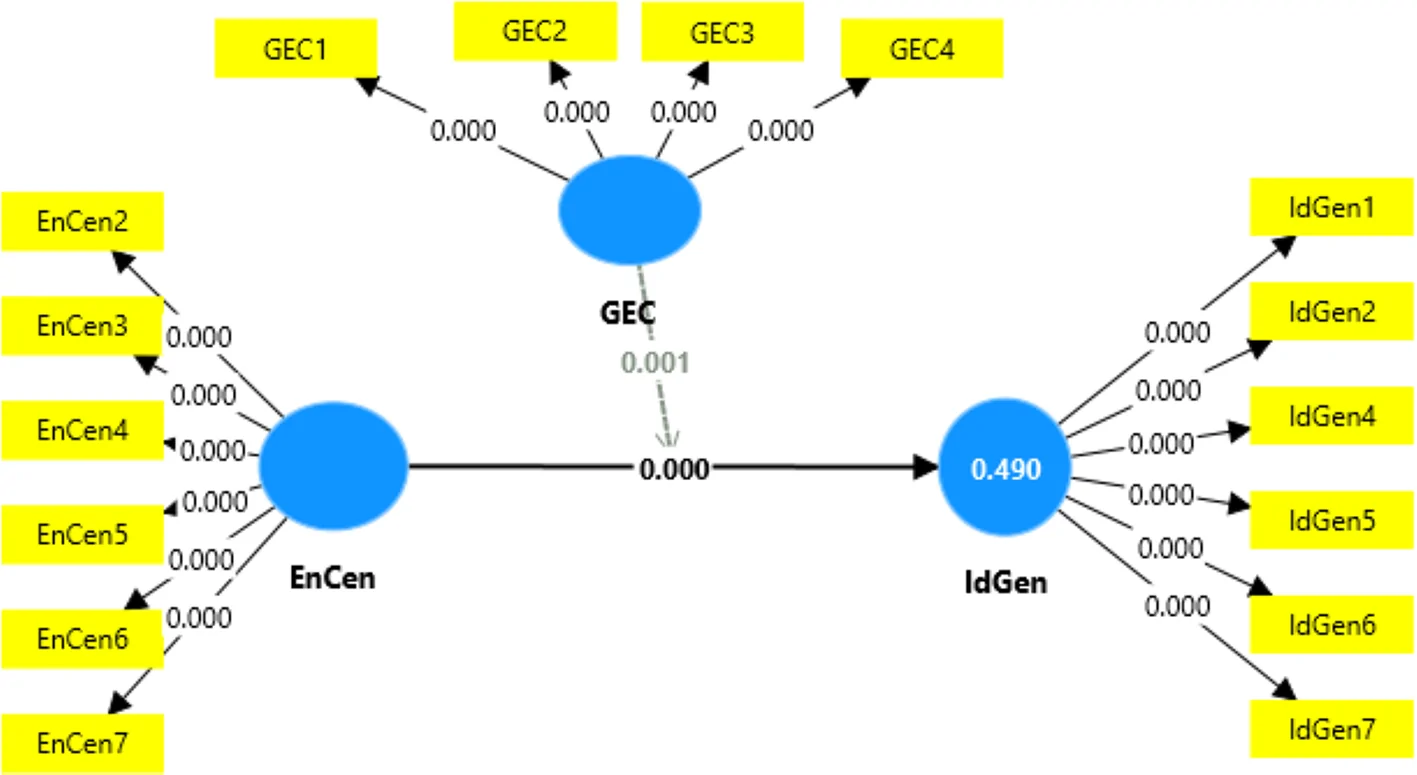
Structural model showing the moderating effect of general economic conditions. This figure displays the structural model incorporating the moderating role of general economic conditions on the link between entrepreneurship centre engagement and idea generation, tested using bootstrapping with 5,000 resamples.

To examine the hypothesised relationships among constructs, the structural model was evaluated using the bootstrapping technique with 5,000 resamples. Figure 3 and Figure 4 present the structural models for both the direct and moderated relationships, respectively.

Table 3 presents the coefficient of determination(R ^2)^ and effect size (F ^2^). The model’s explanatory power was determined using the coefficient of determination (R ^2^). The R ^2^ value for IdGen was 0.416, indicating that approximately 41.6% of the variance in innovative business idea generation was explained by the main effects and the interaction term. According to Ref. 38, this represents a moderate level of explanatory power. Furthermore, the effect size (f ^2^) for EnCen was 0.711, signifying a substantial effect on IdGen.

**Table 3.** Coefficient of determination (R ^2^) and effect size (F ^2^).

R ^2^	R ^2^ Adjusted	F ^2^
0.416	0.413	0.711

### 5.4 Out-of-sample predictive power (Q ^2^) via PLSpredict

To evaluate the predictive validity of the model, the PLSpredict procedure was employed using 10-fold cross-validation, as proposed by Refs. 37, 42 and 44. The predictive accuracy of the PLS model was benchmarked against two reference models: the individual-mean (IA) model and the linear (LM) model. Assessment metrics included Root Mean Squared Error (RMSE), Mean Absolute Error (MAE), and average prediction loss differences. The Q ^2^ value for IdGen was 0.466, indicating moderate-to-strong predictive relevance ( Table 4). The PLS model significantly outperformed the individual-mean (IA) model (p < 0.001) but did not demonstrate superiority over the linear model (LM) in this context.

**Table 4.** Out-of-sample predictive assessment using PLSpredict (Q ^2^).

Construct	Q ^2^_predict	RMSE (PLS)	MAE (PLS)	PLS Loss	IA Loss	LM Loss	Avg Loss Diff (PLS–IA)	t-value (IA)	p-value (IA)	Avg Loss Diff (PLS–LM)	t-value (LM)	p-value (LM)
IdGen	0.466	0.736	0.563	1.069	1.447	1.068	-0.378	4.223	0.000	0.001	0.034	0.973

**N.B**: PLS, IA (individual-mean), and LM (linear model) prediction losses compared using SmartPLS 4.0 PLSpredict algorithm following Hair et al. (2021) and Ringle et al. (2019).

### 5.5 Structural path coefficients and hypothesis testing

The structural model was employed to test the proposed hypotheses, with the results presented in Table 5. All three hypothesised relationships were statistically significant. Engagement with the Entrepreneurship Centre (EnCen) exerted a strong positive effect on Innovative Business Idea Generation (IdGen), reflected by a path coefficient (β) of 0.645. General Economic Conditions (GEC) also demonstrated a significant positive impact on IdGen (β = 0.193). Interestingly, the interaction effect between EnCen and GEC on IdGen was negative and significant (β = –0.148), suggesting a dampening moderation effect of economic challenges on the positive influence of entrepreneurship centre engagement among students.

**Table 5.** Structural model outcome and test of hypotheses.

Hypothesis	Path	β	t-value	p-value	Result
H _1_	EnCen -> IdGen	0.645	17.921	0.000	Supported
H _2_	GEC-> IdGen	0.193	3.369	0.001	Supported
H _3_	EnCen × GEC-> IdGen	-0.148	3.252	0.001	Supported

**Criterion:** β = >.20; t-value = >1.96; P-value = < .005 (Hair *et al*., 2021 & Ringle *et al*., 2019).

Source: Researcher’s Field Result (2025).

The moderation analysis showed that general economic challenges (GEC) significantly moderated the relationship between entrepreneurship centre engagement (EnCen) and innovative business idea generation (IdGen) (β = -0.148, t = 3.252, p = 0.001). Given that both the direct effect of EnCen on IdGen and the interaction effect were statistically significant, this result reflects a partial moderation. This implies that while EnCen positively influences innovative idea generation, its effect is weakened in the presence of adverse economic conditions.

## 6. Discussion

This study examined how engagement with entrepreneurship centres influences innovative business idea generation among students in Nigerian higher education institutions, with general economic conditions (GEC) serving as a moderating variable. The findings provide empirical validation for the critical role of university-based entrepreneurship centres in enhancing student entrepreneurial capacity, particularly in a context where traditional education is often critiqued for its limited alignment with labour market demands.

The significant positive relationship between entrepreneurship centre engagement and innovative business idea generation supports the ecological systems theory by highlighting the influence of immediate institutional environments on individual outcomes. In a country like Nigeria, where the formal education system often struggles with outdated curricula, inadequate practical exposure, and limited access to entrepreneurship-specific infrastructure, the value-added services offered by entrepreneurship centres, such as hands-on training, digital tools, mentorship, and networking, fill a vital gap. These centres function as innovation catalysts within a system still grappling with systemic constraints.

However, the moderation analysis adds a layer of nuance. The interaction between entrepreneurship centre engagement and GEC was negative and significant (β = –0.148, t = 3.252, p = 0.001), suggesting a partial moderation. This implies that although engagement with entrepreneurship centres remains a robust predictor of innovative idea generation, its positive effect diminishes in the face of harsh macroeconomic realities. These realities, including inflation, power instability, insecurity, and restricted access to funding, can undermine even the most well-intentioned institutional efforts.

The implications are critical. While entrepreneurship centres are proving effective, their impact is not immune to the prevailing socio-economic environment. This underlines the interdependence between educational innovation and national development conditions. It also signals the need for multi-stakeholder collaboration to ensure that institutional support is complemented by broader policy and infrastructural reforms. These findings are consistent with prior scholarship, yet they offer a context-specific contribution by showcasing how exosystemic factors, such as economic challenges, exert constraining effects on innovation potential within Nigerian universities.

## 7. Conclusion and implications

This study set out to examine the influence of entrepreneurship centre engagement on innovative business idea generation in Nigerian higher education, with general economic conditions introduced as a moderating variable. The findings reaffirm that entrepreneurship centres play a critical role in bridging the gap between theoretical education and practical entrepreneurial capability. They provide students with resources, mentorship, and real-world exposure that are often lacking in conventional academic programmes.

However, the significant negative interaction between entrepreneurship centre engagement and general economic challenges indicates a partial moderation effect. While entrepreneurship centres have a positive and statistically significant impact on idea generation, their effectiveness is dampened in the face of economic hardship. In the Nigerian context, this is particularly relevant, given the persistent macroeconomic instability that young entrepreneurs face, ranging from high inflation to unreliable power supply and insecurity. These systemic issues hinder the translation of entrepreneurial intent into viable ventures, even when institutional support exists.

### Theoretical implications

By integrating ecological systems theory into the analysis, the study highlights the significance of both institutional (microsystem) and economic (exosystem) influences on students’ entrepreneurial development. The finding of partial moderation provides empirical support for the idea that institutional innovation must be contextualised within its broader socio-economic setting. It contributes to entrepreneurship literature by underscoring the contingent effectiveness of university-based interventions in economically strained environments.

### Practical and institutional implications

For higher education leaders, this study highlights the urgency of institutional resilience. Entrepreneurship centres must go beyond offering standardised training to develop adaptive, resource-efficient models that are responsive to the realities of Nigeria’s economic environment. Institutions should also build stronger linkages with industry, financial institutions, and government agencies to buffer the external shocks that limit student ventures.

University managements should invest in digital infrastructure, mentorship networks, and incubator platforms while lobbying for greater policy alignment and funding from government bodies such as the Tertiary Education Trust Fund (TETFund). By aligning centre activities with national development goals, entrepreneurship centres can also become more strategic and better positioned to attract external funding.

### Policy implications

From a policy perspective, this study underscores the need for a coherent entrepreneurship development framework that integrates educational reform with macroeconomic support. Government agencies must recognise that entrepreneurship development in universities cannot thrive in isolation from broader socio-economic reforms. This includes expanding access to credit facilities, reducing the cost of doing business, and providing stable infrastructure to support student-led enterprises.

### Contextual limitations and transferability

While the study draws from a representative sample of Nigerian university students, the findings may not be directly generalizable to other developing countries with differing educational and economic conditions. However, the underlying theoretical framework remains applicable, and replication studies in other African or global south contexts (typically low- and middle-income nations facing systemic economic and institutional challenges) could help establish broader relevance and comparative insights.

In conclusion, this study affirms that university-based entrepreneurship centres are instrumental in promoting innovation and venture readiness among students in Nigeria. However, their impact is moderated, though not negated, by prevailing economic challenges. Thus, a dual strategy that reinforces institutional interventions with economic policy support is essential for fostering sustainable student entrepreneurship and national development.

## Limitations and suggestions for further research

This study offers meaningful contributions to the discourse on entrepreneurship development in higher education; however, certain limitations must be acknowledged. First, the study is contextually bound to Nigeria, a country with distinct institutional, economic, and infrastructural characteristics. As such, while the findings highlight the importance of entrepreneurship centre engagement and the influence of general economic conditions within this context, caution should be exercised in generalising the results to other countries. Differences in policy environments, institutional support systems, and economic stability may yield different outcomes in other settings.

Second, the cross-sectional research design restricts the ability to establish causal relationships. Future studies adopting longitudinal or experimental designs could provide deeper insights into how sustained entrepreneurship centre engagement influences innovative behaviour over time. Third, the reliance on self-reported data may introduce social desirability bias or inaccuracies in respondent perceptions. Complementary qualitative methods, such as focus group discussions or case studies, could enrich the findings and offer more context-sensitive interpretations.

Additionally, future research should consider exploring other moderating or mediating variables, such as digital literacy, institutional trust, or access to financial support, to uncover broader dynamics that affect idea generation. Studies comparing multiple Nigerian regions or institutions may also provide more localised insights, while comparative studies across countries with similar socio-economic contexts could test the transferability of these findings.

### Consent to participate

All participants voluntarily consented to participate in the study after being informed about its purpose and their rights. No personally identifiable data was collected, and confidentiality was strictly maintained throughout.

### Declaration of funding

This research received no specific grant from any funding agency in the public, commercial, or not-for-profit sectors.

## Copyright

© 2025 Ben-Caleb et al. This work is licensed under a Creative Commons Attribution 4.0 International (CC BY 4.0) license.

## Data Availability

The dataset and extended materials supporting the findings of this study are openly available on Zenodo at https://doi.org/10.5281/zenodo.16403311. ^45^ under a Creative Commons Attribution 4.0 International (CC BY 4.0) license. The upload includes the DATASHARE information file, the structured questionnaire and measurement items used during data collection (Appendix A), as well as supplementary materials comprising the measurement model output tables, including the Fornell-Larcker Criterion and cross-loadings. These materials are sufficient to reproduce the key analyses and support the transparency and replicability of the study. All files are freely accessible and may be reused with an appropriate citation.
