# Innovative Business Idea Generation in Higher Education: The Interplay between Entrepreneurship Centres and Economic Conditions

**DOI:** 10.12688/f1000research.167767.2

**Published:** 2026-07-27

**Authors:** Jane Ogochukwu. Ben-Caleb, Itumeleng Ngowi, Adebanji A.W. Ayeni, Ayandeji Sunday Ayantokun, Inegbedion Henry E., Egbide Ben-Caleb

**Affiliations:** 1 Business Administration, Landmark University College of Business and Social Sciences, Omu-Aran, Kwara, Nigeria; 2 Business Support Studies, Central University of Technology Free State Faculty of Management Sciences, Bloemfontein, Free State, South Africa; 3 Business School, North West University Faculty of Economic and Management Sciences, Potchefstroom, North West, South Africa; 4 Business Administration, Wigwe University, Isiokpo, Rivers State, Nigeria; 5 Business Administration, Bowen University, Iwo, Osun, Nigeria; 6 Accounting and Finance, Landmark University College of Business and Social Sciences, Omu-Aran, Kwara, Nigeria

**Keywords:** Entrepreneurship Education, Entrepreneurship Centres, General Economic Conditions, Ecological Systems Theory, Innovative Ideation, Emerging Economy

## Abstract

**Background:**

Entrepreneurship education is widely promoted as a strategy for reducing graduate unemployment and stimulating innovation in developing economies. Universities are expected to develop entrepreneurial capabilities that enable students to identify and pursue viable opportunities despite economic instability. However, limited empirical evidence explains how entrepreneurship centre engagement influences innovative business idea generation under volatile macroeconomic conditions. Drawing on Ecological Systems Theory, this study examines the effect of entrepreneurship centre engagement on innovative business idea generation among Nigerian undergraduates and investigates the moderating role of perceived general economic conditions. Entrepreneurship centre engagement is conceptualised as a microsystem influence, while general economic conditions represent exosystem forces shaping perceptions of feasibility and opportunity.

**Methods:**

A quantitative cross-sectional design was adopted. Survey data were collected from 269 undergraduate students at a Nigerian university with an established entrepreneurship centre. Data were analysed using Partial Least Squares Structural Equation Modelling to assess direct and moderating effects. Reliability, validity, predictive relevance, and common method bias diagnostics were also examined.

**Results:**

Entrepreneurship centre engagement significantly and positively predicts innovative business idea generation. Perceived general economic conditions also significantly influence ideation. However, the interaction effect is negative and significant, indicating that adverse economic perceptions weaken the positive relationship between entrepreneurship centre engagement and idea generation. The model demonstrates acceptable fit, moderate explanatory power, meaningful effect sizes, and predictive relevance.

**Conclusions:**

The findings show that innovative business idea generation emerges from the interaction between institutional support and broader economic conditions. While entrepreneurship centres strengthen students’ innovative thinking, perceived macroeconomic instability constrains this effect by influencing opportunity evaluation and feasibility perceptions. The study contributes to entrepreneurship education research by demonstrating how macroeconomic conditions shape the ideational outcomes of university-based entrepreneurship engagement in an emerging economy.

## 1. Introduction

Entrepreneurship education has emerged as a strategic response to rising youth unemployment and structural labour market constraints in many developing economies. In Nigeria, persistent graduate unemployment reflects a mismatch between higher education outputs and the absorptive capacity of the formal labour market. ^1^ This challenge has intensified policy attention toward entrepreneurship as a mechanism for employment creation, innovation, and economic participation. Rather than functioning solely as a curricular component, entrepreneurship education is increasingly positioned as a developmental strategy intended to equip graduates with the capacity to identify opportunities, mobilise resources, and create sustainable ventures. ^2^

Recognising the urgency of unemployment pressures, the Nigerian National Universities Commission mandated the integration of entrepreneurship education into tertiary curricula beginning in the 2007 to 2008 academic session. ^3^ This policy reform sought to reposition universities as institutional platforms for venture creation and innovation development. The expectation was that structured exposure to entrepreneurial training would stimulate self employment and reduce dependence on limited salaried opportunities. ^4^ Despite widespread institutional compliance, graduate unemployment remains substantial. ^5^ This persistence suggests that the presence of entrepreneurship courses alone may not sufficiently translate into entrepreneurial outcomes. This poses an important question regarding the mechanisms through which entrepreneurship education is operationalised within universities and the extent to which those mechanisms effectively stimulate entrepreneurial ideation.

To complement classroom instruction, many Nigerian universities have established dedicated entrepreneurship centres. These centres are designed to provide experiential learning environments that simulate real venture processes. They offer structured training programmes, mentorship, incubation support, industry partnerships, innovation competitions, and practical enterprise exposure. ^6^ ^–^ ^8^ Landmark University, for instance, integrates agricultural enterprise programmes and vocational ventures into its entrepreneurship centre activities to immerse students in applied business contexts. Similarly, the University of Lagos and Covenant University operate structured incubation and innovation programmes that combine theoretical knowledge with applied entrepreneurial practice. ^9^ ^–^ ^11^ These developments reflect a broader institutional shift toward embedding entrepreneurship within university ecosystems rather than limiting it to standalone courses.

While these institutional initiatives signal progress, their effectiveness cannot be understood independently of the broader economic environment. Structural economic conditions such as insecurity, inflationary pressures, unstable infrastructure, and constrained access to finance shape how entrepreneurial opportunities are perceived and evaluated. ^12^ ^–^ ^14^ In contexts characterised by macroeconomic volatility, students may perceive higher levels of uncertainty regarding venture feasibility, market stability, and resource availability. Such perceptions influence opportunity recognition, risk assessment, and entrepreneurial confidence. Consequently, institutional support mechanisms may operate differently depending on how students interpret prevailing economic conditions.

Within entrepreneurship literature, innovative business idea generation represents a foundational stage of entrepreneurial behaviour. It refers to the capacity to identify novel opportunities, develop creative solutions to social and market problems, and conceptualise viable business models. ^15^ Universities and entrepreneurship centres play a critical role in stimulating this ideational process through exposure to mentorship, collaborative experimentation, and applied problem solving. ^16^ However, the translation of institutional engagement into sustained innovative ideation may depend on contextual signals that either reinforce or constrain entrepreneurial agency. ^17^

Existing research has examined entrepreneurship education outcomes in general terms, yet important gaps remain. First, empirical attention to entrepreneurship centre engagement as a distinct institutional mechanism remains limited, particularly within the Nigerian higher education context. Second, prior studies rarely integrate macro-level economic conditions into models assessing entrepreneurship education effectiveness. This omission restricts understanding of how institutional interventions interact with broader environmental constraints. Third, few studies adopt a theoretically integrated framework that simultaneously accounts for proximal institutional influences and distal structural conditions in shaping innovative idea generation.

To address these gaps, this study is grounded in Bronfenbrenner’s Ecological Systems Theory. The theory conceptualises human development as occurring within nested and interacting environmental systems. ^18^ Within this framework, entrepreneurship centres constitute a proximal microsystem that directly shapes students’ entrepreneurial learning experiences through mentorship, training, and collaborative exposure. General economic conditions represent influences situated within the exosystem that indirectly shape perceptions of opportunity, risk, and venture feasibility. The macrosystem, reflected in national entrepreneurship education policies, frames institutional priorities and societal expectations regarding entrepreneurial behaviour. By integrating these layers, the ecological perspective provides a coherent explanation of how institutional engagement and structural economic signals jointly influence innovative business idea generation.

Importantly, this study conceptualises general economic conditions through students’ perceptions rather than objective macroeconomic indicators. Entrepreneurial action is often shaped by subjective interpretations of opportunity structures and environmental constraints. Perceived economic instability can influence confidence, opportunity evaluation, and willingness to pursue innovative ideas, even when objective indicators remain constant. Examining perceived economic conditions therefore, aligns with the behavioural foundations of entrepreneurship and allows for a more nuanced understanding of how contextual signals shape entrepreneurial agency.

Against this theoretical and empirical backdrop, this study investigates how engagement with university entrepreneurship centres influences innovative business idea generation among students, and whether this relationship is contingent upon perceived general economic conditions. By empirically testing this interaction within a Nigerian university context, the study contributes to entrepreneurship education literature by integrating institutional engagement mechanisms with macro contextual influences under a unified ecological framework.

Specifically, the study seeks to achieve the following objectives:

i. To examine the influence of entrepreneurship centre engagement on innovative business idea generation among university students.
ii. To assess the direct influence of general economic conditions on innovative business idea generation among university students.
iii. To investigate the moderating role of general economic conditions in the relationship between entrepreneurship centre engagement and innovative business idea generation.

## 2. Literature review and hypotheses development

### 2.1 Entrepreneurship centres as learning ecosystems

Universities have increasingly established entrepreneurship centres to complement classroom-based instruction with experiential environments that simulate real venture processes. These centres provide structured access to mentoring, incubation support, industry engagement, collaborative experimentation, and innovation competitions. ^16^ ^,^ ^19^ ^,^ ^20^ Through repeated participation in these structured activities, students are exposed to opportunity recognition practices, market validation processes, entrepreneurial experimentation, and iterative problem-solving routines that extend beyond theoretical instruction. Such experiential exposure strengthens the cognitive and behavioural foundations required for innovative business idea generation. ^21^ ^,^ ^22^

Ecological Systems Theory provides a coherent framework for explaining how these institutional mechanisms influence entrepreneurial development. The theory posits that individual outcomes emerge through interaction with nested environmental systems. ^18^ The microsystem represents the immediate environment within which individuals directly engage with actors, norms, and resources. Within the university context, entrepreneurship centres function as a microsystem that creates sustained interaction between students and mentors, peers, training structures, and entrepreneurial infrastructure. These repeated and structured interactions shape opportunity perception, experimentation behaviour, and entrepreneurial competence development. The theory therefore, supports the expectation that structured institutional engagement can influence ideational outcomes through direct experiential processes.

Entrepreneurship centre engagement reflects active participation rather than passive exposure. It captures the intensity and frequency with which students interact with mentoring networks, structured training programmes, incubation platforms, venture simulations, and collaborative innovation projects. ^23^ Higher engagement increases experiential learning depth and strengthens creative confidence, opportunity identification capability, and entrepreneurial cognition. Students who frequently receive feedback, test prototypes, and collaborate on venture projects are more likely to generate novel business concepts and refine them into implementable ideas. ^17^ ^,^ ^24^ ^–^ ^26^ Institutional environments that provide access to networks, knowledge diffusion channels, and resource mobilisation structures have been shown to stimulate innovation and socio economic transformation in developing contexts. ^27^ By strengthening relational capital, entrepreneurial self-efficacy, and exposure to applied experimentation, institutional structures catalyse ideation processes and opportunity exploration, university entrepreneurship centres therefore operate as embedded innovation platforms within the academic ecosystem.

Empirical research reinforces this theoretical reasoning. Students who participate in startup competitions, mentoring networks, and incubation programmes report higher levels of entrepreneurial creativity, opportunity recognition, and idea refinement. ^15^ ^,^ ^28^ ^–^ ^30^ Exposure to structured entrepreneurial ecosystems within universities expands knowledge networks and enhances students’ ability to identify feasible market gaps. ^31^ However, the existence of entrepreneurship centres alone does not automatically produce ideational outcomes. The depth of engagement and quality of interaction within the institutional environment determine the magnitude of impact. This distinction underscores the importance of conceptualising entrepreneurship centre engagement as an active microsystem process rather than as a mere institutional presence.

Taken together, theoretical reasoning and empirical evidence suggest that entrepreneurship centre engagement strengthens the cognitive and experiential mechanisms underlying innovative business idea generation. Through sustained interaction within the institutional microsystem, students develop opportunity recognition capabilities, creative competencies, and entrepreneurial confidence that enhance their capacity to conceptualise innovative business ideas. On this basis, the study advances the following hypothesis:

H1: Entrepreneurship centre engagement positively influences innovative business idea generation among university students.

### 2.2 Innovative business idea generation

Entrepreneurial ideation does not occur independently of the broader macroeconomic and institutional environment. Students interpret entrepreneurial opportunities within prevailing economic signals that shape perceptions of feasibility, risk exposure, and expected returns. General economic conditions encompass structural dimensions such as political stability, inflation, infrastructure reliability, gross domestic product growth, access to credit, public debt pressures, regulatory quality, etc. ^32^ ^,^ ^33^ These macro-level forces influence how individuals cognitively evaluate opportunity attractiveness and implementation viability.

Within Ecological Systems Theory, general economic conditions are situated within the exosystem. ^18^ The exosystem consists of external environmental structures that indirectly influence individual behaviour by shaping institutional constraints, resource availability, and opportunity structures. Although students do not directly control macroeconomic variables, their perceptions of economic stability or volatility shape entrepreneurial confidence, opportunity recognition intensity, and feasibility assessments. The ecological framework therefore predicts that macro level signals influence entrepreneurial cognition even when institutional support mechanisms are present. Empirical evidence across diverse contexts confirms that macroeconomic and institutional variables significantly influence entrepreneurial activity. Studies from Euro-area countries show that higher GDP per capita, stronger regulatory quality, and deeper financial markets are positively associated with entrepreneurship, whereas public debt burdens and institutional rigidities can dampen entrepreneurial activity. ^34^ Cross-country evidence further indicates that macroeconomic variables are particularly influential in developing economies, where early-stage entrepreneurial activity is more sensitive to inflationary pressures, financial constraints, and institutional instability. ^35^ Evidence from Nigeria similarly demonstrates that GDP growth and private sector credit stimulate new business creation, while inflation undermines entrepreneurial activity. ^36^

These findings suggest that macroeconomic prosperity and institutional quality enhance opportunity confidence and perceived feasibility, whereas inflation, financial constraints, and structural uncertainty increase perceived risk. However, the literature also recognises a dual dynamic. Economic adversity can trigger necessity driven entrepreneurship in contexts where formal employment opportunities are limited. ^37^ ^–^ ^39^ This duality indicates that macroeconomic conditions may either stimulate or suppress entrepreneurial behaviour depending on how individuals interpret constraints and opportunities.

From a behavioural perspective, perceived general economic conditions influence opportunity recognition and ideational intensity. Students who perceive relative macroeconomic stability may be more willing to experiment with innovative ideas because they anticipate viable market absorption and potential access to financial support. In contrast, students who perceive severe instability may experience cognitive withdrawal due to heightened uncertainty and risk salience, even when institutional resources are available. In developing economies characterised by volatility, economic perceptions may therefore exert a direct influence on innovative business idea generation. Based on this reasoning, the study proposes the following hypothesis:

H2: General economic conditions significantly influence innovative business idea generation among university students.

### 2.3 General economic conditions as a moderating variable

Beyond their direct influence, general economic conditions may shape the strength of the relationship between entrepreneurship centre engagement and innovative business idea generation. Ecological Systems Theory emphasises that environmental systems are interdependent and dynamically interacting. ^18^ Microsystem influences are embedded within broader exosystem structures that can amplify or constrain their effects. This interactional logic provides the theoretical foundation for examining moderation.

Entrepreneurship centres operate within the microsystem by providing mentorship, experiential training, incubation platforms, and collaborative learning environments that stimulate creative experimentation and opportunity recognition. Prior research demonstrates that structured institutional support enhances entrepreneurial cognition, opportunity evaluation, and confidence. ^40^ However, institutional mechanisms do not operate independently of macroeconomic realities.

Comparative international evidence suggests that macroeconomic and institutional quality conditions the strength of entrepreneurial outcomes. ^41^ Studies using Global Entrepreneurship Monitor data reveal that macroeconomic variables exert stronger effects on early-stage entrepreneurship in developing economies than in developed contexts. ^35^ Similarly, research on sustainable entrepreneurship shows that perceived risks and structural barriers significantly influence entrepreneurial behaviour, with government support mechanisms mediating these relationships. ^34^ ^,^ ^37^ These findings indicate that institutional support structures are most effective when aligned with supportive macroeconomic and regulatory environments.

Applying this insight to the university context, the effectiveness of entrepreneurship centre engagement depends partly on how students interpret the surrounding economic environment. When perceived economic conditions are relatively stable, students may experience stronger alignment between institutional encouragement and contextual feasibility signals. In such settings, engagement with entrepreneurship centres may more effectively translate into innovative ideation because institutional scaffolding is reinforced by supportive economic expectations.

In contrast, when students perceive economic instability characterised by inflationary pressure, financial constraints, or infrastructural fragility, macro-level uncertainty may weaken the psychological conversion of institutional learning into ideational confidence. Engagement may still stimulate idea generation, but its magnitude may decline as perceived risk intensifies. Thus, the broader economic environment conditions the strength of the microsystem influence.

This moderation logic extends ecological systems theory by demonstrating that entrepreneurial ideation emerges from the dynamic interaction of institutional engagement and structural economic context. Institutional support does not operate in isolation but is embedded within macroeconomic and regulatory realities that shape perceived opportunity feasibility. By integrating institutional and macroeconomic perspectives into an ecological framework, the study advances understanding of how nested systems jointly influence entrepreneurial cognition in emerging economies. Based on this integrated reasoning, the study advances the following hypothesis:

H3: General economic conditions moderate the relationship between entrepreneurship centre engagement and innovative business idea generation.

## 3. Theoretical framework: Ecological Systems Theory (EST)

This study draws on Urie Bronfenbrenner’s ecological systems theory as a multilevel framework for explaining how individual development emerges from dynamic interaction across nested environmental systems. ^18^ The theory rejects linear explanations of behaviour and instead posits that developmental outcomes result from reciprocal processes between individuals and layered environmental structures. These layers include the microsystem, mesosystem, exosystem, and macrosystem. Each layer operates at a distinct level of proximity to the individual, yet their influences are interdependent and mutually reinforcing.

EST is particularly appropriate for research on entrepreneurship education because it integrates institutional mechanisms and structural economic conditions within a unified explanatory structure. Entrepreneurial thinking among university students cannot be reduced to personality traits or isolated pedagogical methods. It develops through sustained interaction with institutional environments while being shaped by broader socio-economic realities. ^40^ ^,^ ^42^ ^,^ ^43^ This multilevel logic enables examination not only of whether engagement with entrepreneurship centres matters, but also of the contextual conditions under which such engagement becomes more or less effective.

At the most immediate level, the microsystem comprises environments in which individuals engage directly and repeatedly. Within higher education, entrepreneurship centres constitute a central microsystem. These centres provide structured interaction through mentoring, applied training programmes, incubation support, networking platforms, and collaborative venture activities. ^17^ ^,^ ^24^ ^,^ ^32^ ^,^ ^44^ By repeatedly participating in these environments, students gain practical knowledge, improve their ability to recognize opportunities, and boost their creative confidence. EST views these close processes as the main drivers of developmental change. Therefore, engaging with entrepreneurship centers suggests an active way that institutional exposure turns into new business ideas.

The mesosystem captures the interconnections among microsystems. In university contexts, this includes relationships between entrepreneurship centres, academic departments, industry partners, alumni networks, and innovation hubs. These linkages influence the coherence and quality of entrepreneurial learning by facilitating interdisciplinary exchange and knowledge transfer. ^8^ ^,^ ^45^ Although the mesosystem is not directly modelled empirically in this study, it provides the relational architecture that shapes how effectively the microsystem operates.

The exosystem refers to environmental structures that influence individuals indirectly by shaping opportunity structures and perceived constraints. In this study, general economic conditions are conceptualised as exosystem influences. Macroeconomic variables such as inflation, access to finance, infrastructure reliability, regulatory quality, and overall economic stability affect how students evaluate entrepreneurial feasibility and risk. ^43^ ^,^ ^46^ ^,^ ^47^ This study focuses on students’ perceptions of these conditions, recognising that entrepreneurial cognition is driven by subjective interpretation rather than purely objective macroeconomic statistics. Although students do not control macroeconomic forces, their perceptions of stability or volatility shape confidence, risk tolerance, and willingness to develop ideas into actionable concepts. Positioning general economic conditions within the exosystem acknowledges that institutional learning processes operate within broader structural constraints.

The macrosystem represents the wider socio-institutional context that defines cultural norms, regulatory frameworks, and policy priorities. ^48^ ^–^ ^50^ In Nigeria, national directives such as the entrepreneurship education mandate introduced by the National Universities Commission constitute macrosystem influences. These policies shape university mandates, resource allocation patterns, and institutional expectations regarding entrepreneurial outcomes. The macrosystem establishes the normative and regulatory boundaries within which entrepreneurship centres function and within which students interpret entrepreneurial aspirations.

A central proposition of EST is that developmental outcomes emerge from interaction across environmental levels rather than from isolated influences. ^18^ This cross-level interaction provides the theoretical foundation for examining moderation in the present study. Entrepreneurship centre engagement operates within the microsystem as a proximal developmental process. However, the strength of this process may depend on signals emanating from the exosystem. When students perceive economic conditions as stable and supportive, institutional engagement may more strongly translate into innovative ideation because contextual signals reinforce feasibility expectations and opportunity confidence. In contrast, when economic instability is salient, structural uncertainty may weaken the conversion of institutional learning into ideational intensity. The moderation hypothesis therefore reflects an ecological interaction between proximal institutional engagement and broader economic context. By integrating engagement with entrepreneurship centers and perceived economic conditions in a multilevel ecological framework, this study views innovative business idea generation as the result of overlapping and interacting environmental influences. This approach enhances research on entrepreneurship education by moving beyond simple explanations and showing how institutional actions are shaped by structural economic realities.

**Figure 1.**
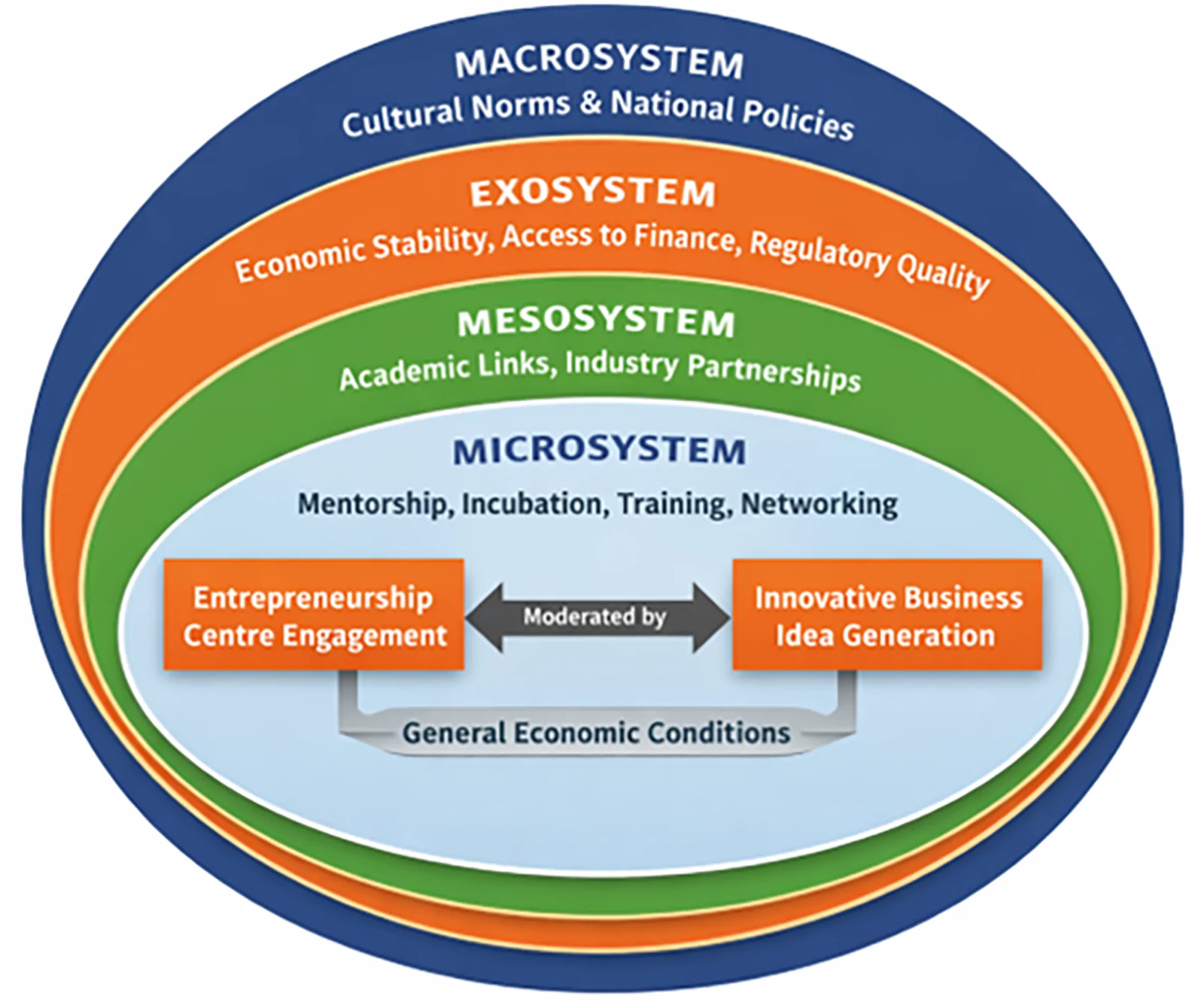
Conceptual framework. N.B: Conceptual model based on the ecological systems theory perspective. **Source:** Researcher’s conceptual framework (2025).

Figure 1 shows the conceptual model based on this ecological perspective.

## 4. Methodology

### 4.1 Research design and paradigm

This study employs a quantitative cross- sectional research design to examine the relationship between entrepreneurship centre engagement and innovative business idea generation, and to assess the moderating role of general economic conditions. The model specifies theoretically derived relationships that require simultaneous estimation of multiple latent constructs. A quantitative design is therefore appropriate because it enables systematic hypothesis testing and structural modelling.

The study is grounded in the positivist paradigm, which assumes that theoretical constructs can be operationalised through measurable indicators and analysed using statistical techniques. This paradigm supports objective hypothesis testing and enhances replicability. The cross-sectional approach captures students’ perceptions and engagement experiences at a defined point in time. While causal inferences are interpreted cautiously, the design is appropriate for examining structural relationships and interaction effects within the ecological framework guiding this study.

### 4.2 Research context and selection

Landmark University was selected as the empirical setting through purposive institutional case selection. The choice was theory-driven. Ecological Systems Theory emphasises the role of clearly structured environmental systems in shaping behavioural outcomes. Landmark University provides an institutionalised entrepreneurship ecosystem that includes a formal entrepreneurship centre, embedded entrepreneurship curriculum, mentoring structures, and venture incubation activities. This makes it an analytically appropriate setting for examining microsystem influences on entrepreneurial ideation.

The focus on a single institutional case enhances internal consistency by limiting contextual heterogeneity. The objective of the study is analytical generalisation grounded in theory rather than statistical generalisation across all Nigerian universities. The selected case, therefore represents a bounded entrepreneurial ecosystem within which ecological interactions can be examined in depth.

### 4.3 Sampling strategy and data collection

The target population consisted of undergraduate students enrolled in Business Administration, Accounting, and Economics programmes. These disciplines were selected because students in business related fields are more likely to engage with entrepreneurship development courses and entrepreneurship centre activities. ^15^ ^,^ ^51^

A purposive sampling technique was adopted to ensure that respondents had meaningful exposure to entrepreneurship education. Eligibility criteria required that participants had completed at least one entrepreneurship development course and had access to the entrepreneurship centre. First year students were excluded because they had not yet completed the required entrepreneurship modules.

Data were collected between February and March 2025 using structured self-administered questionnaires distributed during scheduled academic sessions with institutional approval. Participation was voluntary. Respondents were informed about the purpose of the study, assured of anonymity, and advised that their responses would be used solely for academic research.

A total of 287 questionnaires were distributed. After screening for completeness and response consistency, 269 valid responses were retained for analysis, representing a usable response rate of 93.7 percent.

### 4.4 Sample characteristics

Table 1 presents the demographic characteristics of the respondents. The sample comprised 269 undergraduate students drawn from a Nigerian university with an established entrepreneurship centre. Female students constituted 60.97 percent of the sample, while male students accounted for 39.03 percent. This distribution reflects enrolment patterns within the selected departments and does not suggest systematic sampling imbalance.

**Table 1.** Respondents’ demography.

Variable	Frequency	Percentage (%)
**Gender**		
Male	105	39.03%
Female	164	60.97%
**Age**		
< 20 years	0	0%
20–25 years	269	100%
> 25 years	0	0%
**Department**		
Business Administration	108	40.15%
Accounting	98	36.43%
Economics	63	23.42%
**Total Respondents**	269	100%

**Source:** Researcher’s output (2025).

All respondents were between 20 and 25 years of age, which aligns with the typical age profile of undergraduate students in Nigerian universities. The homogeneity in age enhances comparability across respondents and reduces potential confounding effects associated with generational or life-stage differences in entrepreneurial orientation.

In terms of academic background, 40.15 percent of respondents were enrolled in Business Administration, 36.43 percent in Accounting, and 23.42 percent in Economics. This distribution ensures substantial representation across core business-related disciplines where exposure to entrepreneurship coursework and institutional engagement activities is common. The concentration within business-oriented fields strengthens the analytical relevance of examining entrepreneurship centre engagement and innovative business idea generation, as respondents are situated within programmes that formally integrate entrepreneurial learning components.

The study’s demographic composition reflects a focused and academically relevant sample appropriate for examining institutional engagement processes within a structured university-based entrepreneurship ecosystem.

### 4.5 Sample size justification

Sample adequacy was assessed using established guidelines for Partial Least Squares Structural Equation Modelling. The structural model includes two direct predictors and one interaction term directed at a single endogenous construct. Following recommended minimum sample guidelines, the required sample size is substantially lower than the 269 observations obtained in this study.

In addition, statistical power analysis was conducted using the G Power procedure for multiple regression with three predictors. Assuming a medium effect size, a significance level of 0.05, and desired statistical power of 0.80, the estimated minimum sample size was 92 respondents. The final sample therefore exceeds recommended thresholds and provides sufficient power to detect meaningful structural relationships.

### 4.6 Ethical approval

This study received ethical clearance from the Landmark University Institutional Research Ethics Committee (LMUIREC) under Ref. No: LMUIREC/HS/031/2025, dated 09–06–2025. The protocol was reviewed and approved under the oversight of the Landmark University Centre for Research Innovations and Discoveries (LUCRID). All data collection procedures adhered to the university’s ethical guidelines, and informed consent was obtained from all participants.

### 4.7 Instruments and measures

All constructs were operationalised as reflective latent variables using measurement items adapted from prior validated studies to ensure theoretical grounding and content validity. It measured three latent constructs where EnCen is the independent variable, IdGen (dependent variable) and GEC (moderating variable).

**Entrepreneurship Centre Engagement (EnCen):**

This was measured using items adapted from studies examining university-based entrepreneurship ecosystems and institutional engagement mechanisms. ^8^ ^,^ ^16^ ^,^ ^52^ The scale captures participation in training programmes, mentoring sessions, incubation initiatives, and utilisation of entrepreneurial support resources. A sample statement is “I regularly participate in training sessions offered by the entrepreneurship centre”.

**Innovative Business Idea Generation (IdGen):**

Assessed creative thinking, opportunity recognition, idea articulation, and novelty of business concepts from previously validated scales. ^21^ ^,^ ^22^ ^,^ ^53^ A sample statement is “I frequently come up with new business ideas”.

**General Economic Conditions (GEC):**

General Economic Conditions was measured using perception based macroeconomic indicators capturing subjective evaluations of inflationary pressure, financial access constraints, infrastructural reliability, and economic stability. ^46^ ^,^ ^54^ ^,^ ^55^ A perception based operationalisation is theoretically consistent with ecological systems theory because exosystem influences operate through individual interpretation of structural conditions. A sample statement is “Rising inflation and the cost of living make it difficult to finance a business idea”.

All items were rated using a five-point Likert scale (1 = Strongly Disagree to 5 = Strongly Agree). A pilot study involving 25 students was conducted to assess clarity, internal consistency, and contextual appropriateness, leading to minor refinements. Details of the scale items are presented in Appendix A.

### 4.8 Procedures for addressing common method bias

Because data were collected using a single self-administered questionnaire, both procedural and statistical strategies were incorporated to minimise the risk of common method bias.

At the design stage, several procedural remedies were implemented. Respondents were assured of anonymity and confidentiality in order to reduce evaluation apprehension and social desirability tendencies. The questionnaire included clear instructions and neutral wording to minimise ambiguity and item misinterpretation. Predictor and criterion constructs were presented in separate sections of the instrument to reduce the likelihood of response pattern consistency. In addition, scale endpoints were clearly defined and varied conceptually across constructs to reduce mechanical answering behaviour. At the analytical stage, statistical assessments were conducted to evaluate the potential presence of common method variance. These diagnostic procedures are presented and discussed in the Results section.

### 4.9 Data analysis technique

Data were analysed using Partial Least Squares Structural Equation Modeling (PLS-SEM) in SmartPLS 4.1.1.7. ^56^ This method was selected for its suitability for predictive research, theory development, and models incorporating interaction effects. The method does not require multivariate normality and is appropriate for complex models with multiple latent constructs. ^57^ ^,^ ^58^

Model evaluation proceeded in two stages, as recommended by. ^58^ In the first stage, the measurement model evaluation focused on indicator reliability, internal consistency reliability, convergent validity, and discriminant validity. Indicator reliability was assessed using standardized outer loadings, with values of 0.70 or higher indicating acceptable reliability for reflective constructs. Internal consistency reliability was examined using Cronbach’s alpha, rhoA, and composite reliability. Values above 0.70 were considered satisfactory. Convergent validity was assessed using the Average Variance Extracted, where values above 0.50 indicate that constructs explain more than half of the variance in their indicators.

Discriminant validity was evaluated using the heterotrait–monotrait ratio of correlations. Values below the conservative threshold of 0.85 indicate adequate construct distinctiveness. ^59^ The Fornell–Larcker criterion and cross-loadings were also examined to provide additional evidence of discriminant validity.

After establishing measurement adequacy, the structural model was evaluated. This involved a bootstrapping procedure with 5,000 resamples to estimate the path coefficients, effect sizes (f ^2^), and statistical significance of each relationship. The model’s explanatory power was then evaluated using the coefficient of determination (R ^2^) to determine the proportion of variance explained in the dependent variable, and predictive relevance (Q ^2^) was assessed to confirm the model’s capability in forecasting outcomes.

Additional diagnostic assessments included collinearity testing using variance inflation factors, common method bias assessment using full collinearity VIF, common method bias, model fit indices including SRMR and NFI, and predictive relevance using PLSpredict. The PLSpredict algorithm was applied to generate out-of-sample predictive performance. The model’s Root Mean Square Error (RMSE) and Mean Absolute Error (MAE) were benchmarked against linear regression and mean-based models. ^60^ ^,^ ^61^

The study utilised sixteen indicators across the three constructs, with all statistical thresholds meeting acceptable benchmarks for reliability and validity.

## 5. Results

### 5.1 Preliminary data diagnostics

The final dataset consisted of 269 valid observations with no missing values. Initial data screening confirmed completeness and suitability for Partial Least Squares Structural Equation Modelling. Descriptive statistics and collinearity diagnostics indicated no violations of PLS-SEM assumptions.

Indicator reliability was examined using standardized outer loadings. Two indicators did not meet the recommended thresholds for reflective measurement models. EnCen1 exhibited a loading of 0.188, indicating inadequate convergence with its latent construct. IdGen3 showed a loading of 0.596, which falls below the commonly accepted minimum threshold of 0.60 for exploratory contexts and below the preferred 0.70 benchmark for established constructs. ^58^ ^,^ ^60^

**Figure 2.**
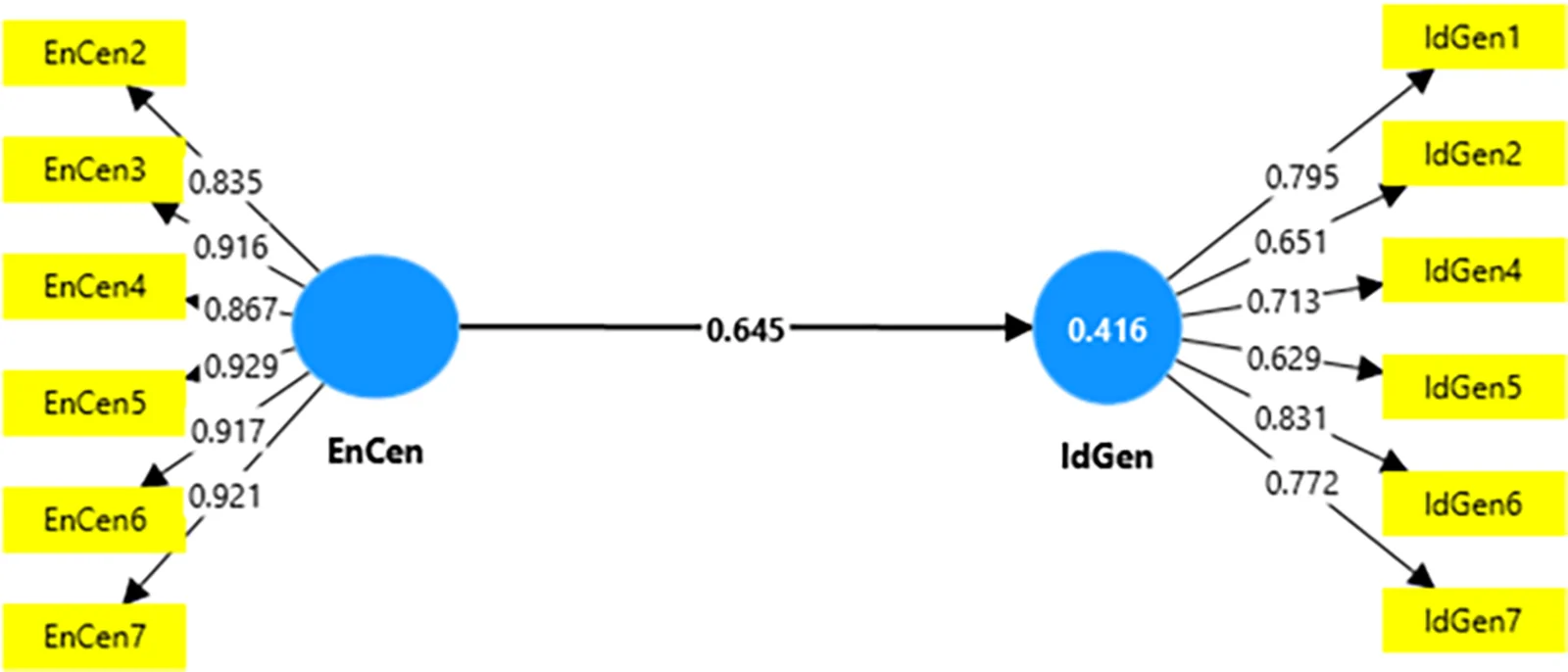
Indicator loadings. **Source:** Researcher’s output (2025). This figure presents the final indicator loadings results after excluding EnCen1 and IdGen3 due to low factor loadings (< 0.60). Indicator loadings and R ^2^ values shows enhanced internal consistency and convergent validity.

Consistent with PLS-SEM guidelines, indicators with very low loadings were removed to improve measurement quality. The exclusion of EnCen1 and IdGen3 enhanced internal consistency and convergent validity without compromising the conceptual domain of the constructs, as the remaining indicators adequately captured the theoretical dimensions of entrepreneurship centre engagement and innovative business idea generation. The refined model was subsequently re-estimated using the retained indicators. Final indicator loadings results are presented in Figure 2.

### 5.2 Measurement model evaluation

The measurement model was assessed using established PLS-SEM criteria, including indicator reliability, internal consistency reliability, convergent validity, and discriminant validity.

Indicator reliability was evaluated using standardized outer loadings and bootstrapped significance levels. As shown in Table 2, all retained indicators exhibit loadings above 0.70, indicating satisfactory convergence with their respective constructs. All loadings are statistically significant at p < 0.001, with confidence intervals that do not include zero, confirming indicator stability.

**Table 2.** Outer loadings and significance of measurement items.

Indicator	Construct	Loading	t-value	p-value	95% CI (LL–UL)
EnCen2	Entrepreneurship Centre Engagement	0.826	28.357	0.000	0.760–0.875
EnCen3	Entrepreneurship Centre Engagement	0.912	63.234	0.000	0.883–0.939
EnCen4	Entrepreneurship Centre Engagement	0.871	44.886	0.000	0.833–0.907
EnCen5	Entrepreneurship Centre Engagement	0.932	79.683	0.000	0.908–0.954
EnCen6	Entrepreneurship Centre Engagement	0.921	69.955	0.000	0.895–0.946
EnCen7	Entrepreneurship Centre Engagement	0.921	70.589	0.000	0.895–0.946
GEC1	General Economic Conditions	0.883	42.160	0.000	0.842–0.922
GEC2	General Economic Conditions	0.859	33.067	0.000	0.810–0.906
GEC3	General Economic Conditions	0.867	34.523	0.000	0.818–0.914
GEC4	General Economic Conditions	0.809	24.760	0.000	0.748–0.865
IdGen1	Innovative Business Idea Generation	0.735	15.516	0.000	0.645–0.817
IdGen2	Innovative Business Idea Generation	0.798	22.624	0.000	0.725–0.866
IdGen4	Innovative Business Idea Generation	0.820	21.230	0.000	0.745–0.887
IdGen5	Innovative Business Idea Generation	0.771	19.820	0.000	0.696–0.839
IdGen6	Innovative Business Idea Generation	0.813	24.386	0.000	0.747–0.876
IdGen7	Innovative Business Idea Generation	0.790	20.925	0.000	0.714–0.855

**Source:** Researcher’s output (2025).

The removal of EnCen1 and IdGen3 improved the overall quality of the measurement model. The remaining indicators demonstrate strong empirical association with their constructs, supporting adequate construct representation. These results confirm satisfactory indicator reliability for Entrepreneurship Centre Engagement, General Economic Conditions, and Innovative Business Idea Generation.

Table 2 presents the final outer loadings, t-values, p-values, and confidence intervals for all retained indicators.

Internal consistency reliability and convergent validity were assessed using Cronbach’s alpha, rhoA, composite reliability (CR), and average variance extracted (AVE). As shown in Table 3, all constructs exceeded recommended threshold values (α > 0.70, CR > 0.70, AVE > 0.50). ^60^ Entrepreneurship Centre Engagement demonstrated excellent internal consistency, with Cronbach’s alpha of 0.953, rhoA of 0.954, and composite reliability of 0.961. General Economic Conditions exhibited strong reliability (α = 0.859; CR = 0.905), while Innovative Business Idea Generation also showed satisfactory internal consistency (α = 0.875; CR = 0.906).

**Table 3.** Reliability and convergent validity.

Construct	Cronbach’s alpha	rhoA	Composite reliability	AVE
Entrepreneurship Centre Engagement	0.953	0.954	0.961	0.806
General Economic Conditions	0.859	0.861	0.905	0.705
Innovative Business Idea Generation	0.875	0.877	0.906	0.616

**Source:** Researcher’s output (2025).

The AVE values ranged from 0.616 to 0.806, exceeding the 0.50 benchmark. This indicates that each construct explains more than 50% of the variance in its indicators, thereby confirming adequate convergent validity.

Discriminant validity was evaluated using the heterotrait–monotrait ratio (HTMT) of correlations, consistent with current best practice in PLS-SEM as recommended by. ^60^ ^,^ ^62^ As reported in Table 4a, all HTMT values were below the conservative threshold of 0.85, indicating satisfactory construct distinctiveness. Furthermore, bootstrapped confidence intervals did not include the value of 1.00, providing additional statistical support for discriminant validity.

**Table 4a.** Discriminant validity – HTMT ratios.

Constructs	HTMT
EnCen – IdGen	0.786
EnCen – GEC	0.180
IdGen – GEC	0.296

**Source:** Researcher’s output (2025).

**Table 4b.** Discriminant validity – Fornell–Larcker criterion.

Construct	EnCen	GEC	IdGen
EnCen	**0.898**		
GEC	0.158	**0.840**	
IdGen	0.641	0.271	**0.785**

Diagonal elements represent square root of AVE.

**Source:** Researcher’s output (2025).

The Fornell–Larcker criterion was also examined as a complementary assessment. As shown in Table 4b, the square root of the AVE for each construct exceeded its corresponding inter-construct correlations. Cross-loading analysis further confirmed that each indicator loaded highest on its associated construct relative to other constructs. These results confirm adequate discriminant validity of the measurement model.

### 5.3 Structural model evaluation

**Collinearity and common method bias assessment**

Prior to hypothesis testing, collinearity diagnostics were examined to ensure that the estimated structural relationships were not distorted by multicollinearity. The structural (inner) variance inflation factor (VIF) values were 1.026 for Entrepreneurship Centre Engagement (EnCen), 1.212 for General Economic Conditions (GEC), and 1.236 for the interaction term (EnCen × GEC), all of which fall well below the conservative threshold of 3.3 ( Table 6). These findings indicate that multicollinearity does not threaten the stability or interpretation of the structural model estimates. At the indicator level, outer VIF values were also assessed for completeness. Although some indicator VIFs exceeded 3.0, all remained below the conservative threshold of 5.0 ( Table 7), suggesting the absence of critical multicollinearity concerns among the measurement items.

**Table 5.** Harman’s single-factor test.

Component	Variance explained (%)	Threshold	Conclusion
First Factor	32.4%	< 50%	No evidence of dominant single-factor bias

**Table 6.** Collinearity assessment – Inner model (Structural VIF).

Path	VIF
EnCen - > IdGen	1.026
GEC- > IdGen	1.212
EnCen × GEC - > IdGen	1.236

**Source:** Researcher’s output (2025).

**Table 7.** Indicator (Outer) VIF.

Indicator	VIF
EnCen2	2.135
EnCen3	3.928
EnCen4	2.890
EnCen5	4.484
EnCen6	3.965
EnCen7	3.989
GEC1	2.851
GEC2	2.315
GEC3	2.375
GEC4	1.773
IdGen1	1.944
IdGen2	2.601
IdGen4	2.730
IdGen5	2.226
IdGen6	2.703
IdGen7	2.430

**Source:** Researcher’s output (2025).

Given the cross-sectional and self-reported nature of the data, common method bias (CMB) was further assessed using both Harman’s single-factor test and the full collinearity VIF approach. The unrotated principal component analysis revealed multiple factors with eigenvalues greater than one, while the first factor accounted for 32.4% of the total variance, which is below the commonly accepted 50% threshold for detecting substantial common method variance ( Table 5). In addition, all structural VIF values were below 3.3. These results suggest that common method variance is unlikely to inflate the observed relationships substantially. Combined with the procedural remedies implemented during questionnaire design and administration, the findings indicate that the study’s results are not artefacts of single-source data collection.

**Coefficient of determination and effect size**

The model explained 48.8% of the variance in Innovative Business Idea Generation (R ^2^ = 0.488; adjusted R ^2^ = 0.482). According to, ^57^ ^,^ ^59^ this value indicates moderate explanatory power. This suggests that entrepreneurship centre engagement and perceived general economic conditions jointly account for a substantial proportion of variance in students’ innovative ideation. Importantly, the inclusion of the interaction term increased the R ^2^ relative to the direct-effects-only model, indicating that macroeconomic perceptions provide additional explanatory value beyond institutional engagement alone. This improvement supports the theoretical proposition that entrepreneurial ideation emerges from the interaction between microsystem engagement and exosystem economic context.

The effect size (f ^2^) results further clarify the substantive contributions of each predictor. Entrepreneurship Centre Engagement demonstrates a large effect size (f ^2^ = 0.621), exceeding the conventional threshold of 0.35 (Cohen, 1988). This indicates strong practical relevance and confirms that institutional engagement is the primary driver of innovative business idea generation within the study context. Perceived General Economic Conditions exhibit a moderate effect size (f ^2^ = 0.193), suggesting that broader economic perceptions independently influence ideational outcomes. The interaction effect between Entrepreneurship Centre Engagement and General Economic Conditions shows a small to moderate effect size (f ^2^ = 0.061), exceeding the minimum threshold of 0.02. While the moderation effect is comparatively smaller, it remains substantively meaningful, indicating that economic perceptions condition the strength of institutional engagement effects rather than displacing them.

Put together, the pattern of effect sizes reinforces the central role of entrepreneurship centre engagement while providing empirical support for a contextual conditioning effect of perceived macroeconomic conditions. Table 8 presents the detailed results for both the coefficient of determination (R ^2^) and effect sizes (f ^2^).

**Table 8.** Coefficient of determination (R ^2^) and effect size (f ^2^) summary.

Endogenous construct	R ^**2**^	Adjusted R ^2^	Structural path	f ^2^
Innovative Business Idea Generation	0.488	0.482	EnCen → IdGen	0.621
			GEC → IdGen	0.193
			EnCen × GEC → IdGen	0.061

**Source**: Researcher’s output (2025).

### 5.4 Model fit assessment

Global model fit indices were examined to enhance reporting transparency, although Partial Least Squares Structural Equation Modelling is primarily oriented toward prediction rather than covariance-based fit evaluation. ^63^ ^,^ ^64^

The Standardized Root Mean Square Residual (SRMR) was 0.093. Although slightly above the conservative 0.08 guideline, recent PLS-SEM literature cautions against rigid application of covariance-based cut-offs in variance-based models, particularly in moderation frameworks and prediction-oriented studies. ^60^ Given the satisfactory explanatory power, effect sizes, predictive relevance, and absence of collinearity issues, the model fit is considered acceptable for theory development purposes.

The Normed Fit Index (NFI) was 0.734, suggesting moderate model fit. Although below the stricter 0.90 benchmark commonly applied in covariance-based SEM, ^59^ caution that traditional global fit thresholds should not be rigidly imposed on PLS-SEM models, particularly when the objective is theory development and prediction rather than model reproduction.

The chi-square value is reported for completeness; however, consistent with PLS-SEM guidance, inferential emphasis is placed on explanatory power, predictive relevance, and effect sizes rather than covariance-based discrepancy statistics.

Table 9 display the model demonstrates acceptable fit for a prediction-oriented structural model situated within an emerging research context.

**Table 9.** Model fit indices.

Fit index	Value
SRMR	0.093
NFI	0.734
Chi-Square	701.699

**Source:** Researcher’s output (2025).

**Out-of-Sample Predictive Power (Q ^2^) via PLSpredict**

To assess predictive validity, the PLSpredict procedure was implemented using 10-fold cross-validation, following the guidelines of. ^56^ ^,^ ^61^ ^,^ ^65^ This approach evaluates out-of-sample predictive performance by comparing the PLS-SEM model against two benchmark models: the naïve individual-mean (IA) model and a linear regression (LM) model. Predictive accuracy was examined using Q ^2^predict, root mean squared error (RMSE), mean absolute error (MAE), and cross-validated prediction loss differences.

Table 10 shows that Innovative Business Idea Generation achieved a Q ^2^predict value of 0.479, indicating substantial out-of-sample predictive relevance. The positive Q ^2^predict value confirms that the model generates more accurate predictions than a simple mean-based benchmark. The RMSE (0.684) and MAE (0.527) values further indicate stable prediction error levels for the endogenous construct.

**Table 10.** Out-of-sample predictive assessment using PLSpredict (Q ^2^).

Construct	Q ^2^ predict	RMSE (PLS)	MAE (PLS)
Innovative Business Idea Generation	0.479	0.684	0.527

**Source**: Researcher’s output (2025).

The Cross-Validated Predictive Ability Test (CVPAT) results presented in Table 11 provide additional evidence of predictive performance. The PLS model significantly outperformed the individual-mean benchmark (loss difference = −0.334, t = 4.007, p < 0.001), demonstrating superior predictive accuracy relative to a naïve model. However, no statistically significant difference was observed between the PLS model and the linear regression model (loss difference = 0.008, t = 0.193, p = 0.847), indicating comparable predictive performance.

**Table 11.** Cross-validated prediction loss (CVPAT).

Comparison	Loss difference	t-value	p-value
PLS vs IA	−0.334	4.007	0.000
PLS vs LM	0.008	0.193	0.847

**Source**: Researcher’s output (2025).

These findings confirm that the structural model possesses meaningful out-of-sample predictive capability. The results support the practical utility of the proposed framework for forecasting innovative business idea generation within an emerging economy context.

### 5.5 Structural path coefficients and hypothesis testing

Path coefficients were estimated using the PLS algorithm, and statistical significance was assessed through a bootstrapping procedure with 5,000 resamples. Bias-corrected confidence intervals were examined to confirm the robustness of the estimated relationships.

Model evaluation proceeded in two stages. First, the direct structural relationships between entrepreneurship centre engagement and general economic conditions on innovative business idea generation were estimated. Second, the moderation model was specified by incorporating the interaction term between entrepreneurship centre engagement and general economic conditions using the two-stage approach.

The moderation model explains 48.8% of the variance in innovative business idea generation (r ^2^ = 0.488; adjusted r ^2^ = 0.482), indicating moderate explanatory power. The inclusion of the interaction term provided incremental explanatory value relative to the direct-effects-only specification, supporting the theoretical relevance of modelling cross-level interaction.

As reported in Table 12, entrepreneurship centre engagement exerts a strong and statistically significant positive effect on innovative business idea generation (β = 0.558, t = 6.145, p < 0.001). The 95% confidence interval [0.387, 0.736] excludes zero, confirming the robustness of the relationship. The associated effect size is large (f ^2^ = 0.621), establishing entrepreneurship centre engagement as the dominant predictor in the model. Thus, **H**_**1**_ is supported.

**Table 12.** Hypothesised paths and structural model results.

Hypothesis	Path	β	t-value	p-value	95% CI	f ^**2**^	Decision
**H**_**1**_	EnCen → IdGen	0.558	6.145	0.000	0.387–0.736	0.621	Supported
**H**_**2**_	GEC → IdGen	0.193	3.667	0.000	0.139–0.456	0.193	Supported
**H**_**3**_	EnCen × GEC → IdGen	−0.146	3.188	0.001	−0.237 – −0.057	0.061	Supported

**Source**: Researcher’s output (2025).

General Economic Conditions also demonstrate a positive and statistically significant direct effect on Innovative Business Idea Generation (β = 0.193, t = 3.667, p < 0.001). The confidence interval [0.139, 0.456] excludes zero, and the effect size (f ^2^ = 0.193) indicates moderate practical relevance. Accordingly, **H**_**2**_ is supported.

The interaction term between entrepreneurship centre engagement and general economic conditions is negative and statistically significant (β = −0.146, t = 3.188, p = 0.001), the 95% confidence interval [−0.237, −0.057] does not include zero, confirming the presence of moderation. The effect size (f ^2^ = 0.061) indicates a small to moderate interaction effect. This finding demonstrates that adverse economic perceptions weaken the positive relationship between institutional engagement and innovative ideation. Thus, **H**_**3**_ is supported.

These results indicate that while entrepreneurship centre engagement remains the dominant driver of innovative business idea generation, its effectiveness is conditioned by macroeconomic perceptions.

**Moderation plot and simple slope interpretation**

**Figure 3.**
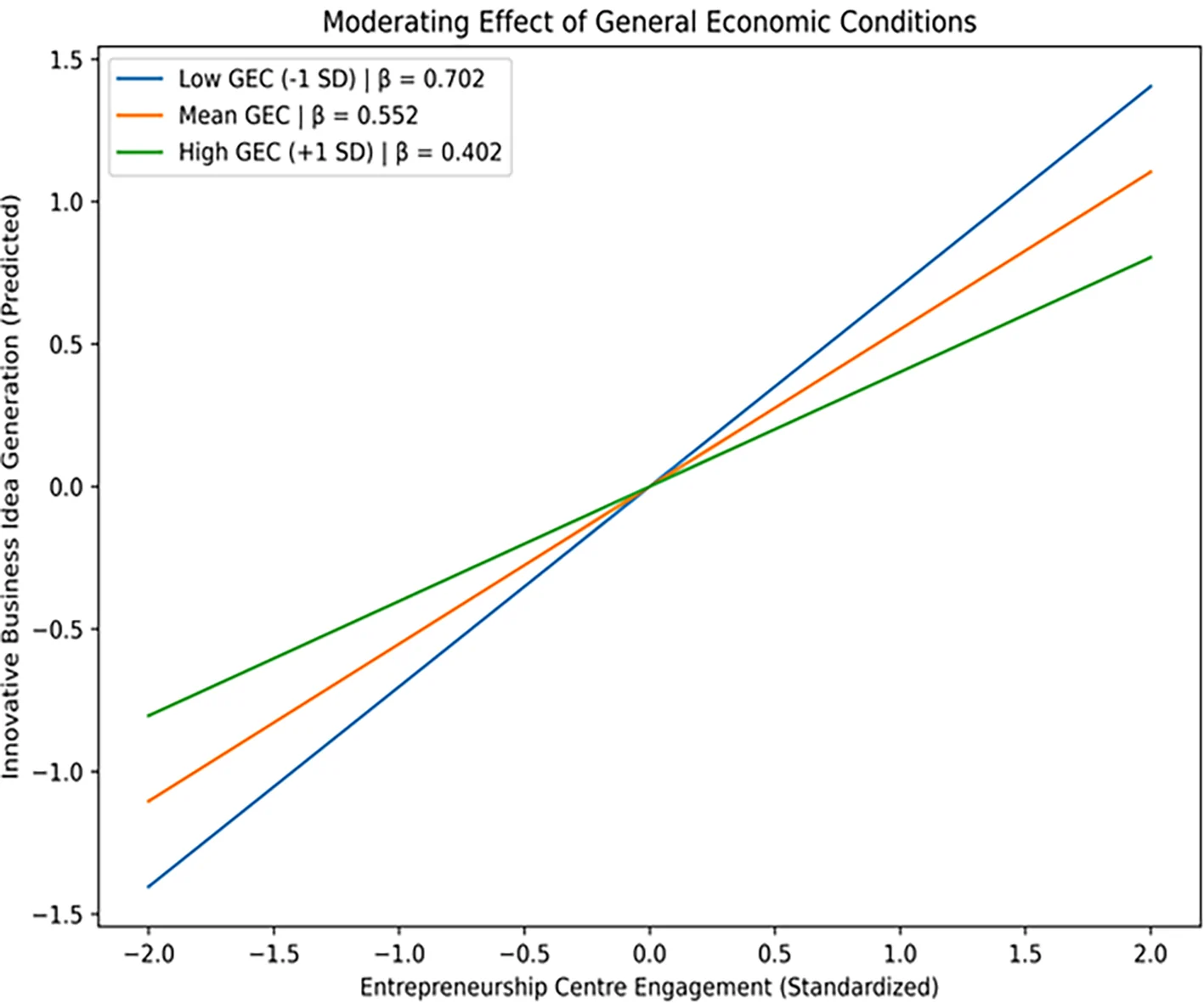
Simple slop plot. **Source**: Researcher’s output (2025).

The moderation effect of General Economic Conditions was further examined using a simple slope analysis. Figure 3 illustrates three conditional effects corresponding to low (−1 SD), mean, and high (+1 SD) levels of perceived economic conditions. This graphical representation clarifies how the strength of the relationship between Entrepreneurship Centre Engagement and Innovative Business Idea Generation varies across economic contexts.

The conditional effects indicate that when perceived economic constraints are low, the positive association between entrepreneurship centre engagement and innovative idea generation is strongest (β = 0.702). At the mean level of perceived economic conditions, the relationship remains positive but weaker (β = 0.552). At high levels of perceived economic constraints, the magnitude of the relationship declines further (β = 0.402).

These results demonstrate that entrepreneurship centre engagement consistently promotes innovative ideation across all economic contexts; however, its strength diminishes as perceptions of macroeconomic instability intensify. The statistically significant negative interaction term (β = −0.148, p = 0.001) confirms a dampening moderation effect. Adverse economic perceptions do not reverse the positive association but attenuate its magnitude.

Substantively, this pattern suggests that entrepreneurship centres remain effective institutional mechanisms for fostering innovation, yet their impact is partially constrained when students perceive the broader economic environment as unstable. The findings reinforce the cross-level ecological argument that institutional processes operate within, and are conditioned by, macroeconomic structures.

**Comparison of direct and moderated models**

To assess the incremental contribution of the moderating mechanism, a comparative analysis was conducted between the direct-only structural model and the moderated model incorporating General Economic Conditions and the interaction term.

Table 13 shows that in the direct-only model, Entrepreneurship Centre Engagement exhibited a strong positive effect on Innovative Business Idea Generation (β = 0.645, t = 17.921, p < 0.001), explaining 41.6% of the variance (R ^2^ = 0.416). The associated effect size was large (f ^2^ = 0.711), confirming engagement as a dominant institutional predictor when considered independently.

**Table 13.** Comparison of direct-only and moderated structural models.

Model specification	Direct-only model	Moderated model
R ^2^ (IdGen)	0.416	0.488
R ^2^ Adjusted	0.413	0.482
ΔR ^2^	–	+0.072
EnCen - > IdGen (β)	0.645	0.552
t-value	17.921	(see bootstrapping output)
p-value	< 0.001	< 0.001
f ^2^ (EnCen)	0.711 (large)	0.576 (large)
GEC - > IdGen (β)	–	0.193
f ^2^ (GEC)	–	0.059 (small)
Interaction (GEC × EnCen) β	–	−0.150
f ^2^ (Interaction)	–	0.062 (small–moderate)
Inner VIF (EnCen)	1.000	1.032
Inner VIF (GEC)	–	1.299
Inner VIF (Interaction)	–	1.288
SRMR	0.091	0.093
NFI	0.870	0.860

**Source:** Researcher’s Output (2025).

With the inclusion of General Economic Conditions and the interaction term, the explanatory power increased to R ^2^ = 0.488, representing an additional 7.2% of explained variance (ΔR ^2^ = 0.072). The direct effect of Entrepreneurship Centre Engagement decreased to β = 0.552 but remained statistically significant, while the interaction term was negative and significant (β = −0.150) with a small-to-moderate effect size (f ^2^ = 0.062). This pattern indicates partial moderation.

The reduction in the direct coefficient alongside the significant interaction confirms that the effectiveness of entrepreneurship centre engagement is contingent upon perceived economic conditions. Effect size comparisons show that although engagement remains the primary driver of ideation, macroeconomic perception contributes meaningful explanatory value. Collinearity diagnostics indicate no instability introduced by the moderator, as all inner VIF values remain well below conservative thresholds. Fit indices remain comparable across model specifications.

## 6. Discussion

This study examined how engagement with university entrepreneurship centres influences innovative business idea generation and how this relationship is conditioned by perceived general economic conditions. The findings provide robust empirical evidence that institutional engagement plays a central developmental role in shaping entrepreneurial ideation while revealing how macroeconomic perceptions condition the strength of this influence.

The results show that entrepreneurship centre engagement is a strong and substantively meaningful predictor of innovative business idea generation. This finding aligns with prior research demonstrating that experiential and ecosystem-based entrepreneurship education enhances opportunity recognition and venture intention. ^21^ ^,^ ^22^ ^,^ ^32^ ^,^ ^66^ ^,^ ^67^ The large effect size indicates that sustained participation in mentoring, incubation, collaborative experimentation, and experiential training significantly strengthens students’ ideational capacity. This finding reinforces the argument that entrepreneurship education becomes transformative when it extends beyond formal classroom instruction into structured institutional ecosystems. Repeated interaction with mentors, peers, and venture support platforms appears to enhance opportunity recognition, creative confidence, and cognitive flexibility. Entrepreneurial ideation therefore, emerges as a developmental outcome of structured engagement rather than merely an individual predisposition.

In addition, from the perspective of ecological systems theory, this confirms the primacy of the microsystem in shaping developmental processes. Entrepreneurship centres function as proximal environments in which iterative interaction drives cognitive and behavioural transformation. This finding responds directly to critiques that entrepreneurship education in developing economies is excessively theoretical and insufficiently practice-oriented. ^3^ ^,^ ^4^ ^,^ ^31^ The evidence suggests that when institutional engagement is substantive and experiential, it can meaningfully stimulate innovative thinking among students.

Furthermore, the results demonstrate that perceived general economic conditions exert a significant direct influence on innovative idea generation. Students who interpret the broader economic environment as stable and supportive report stronger ideational outcomes. This suggests that macroeconomic perceptions shape opportunity confidence and perceived feasibility. Economic signals influence whether students interpret entrepreneurial ideas as actionable opportunities or as distant aspirations. This supports prior macro-entrepreneurship research showing that stable institutional environments foster opportunity confidence. ^27^ ^,^ ^33^ ^,^ ^37^ However, unlike prior national-level studies, the present study demonstrates this effect within a university-based ideational process. ^34^ ^,^ ^35^ ^,^ ^38^

Also, the findings from the moderation provide deeper theoretical insight. The negative interaction effect indicates that adverse economic perceptions weaken the positive association between entrepreneurship centre engagement and innovative business idea generation. Although engagement remains beneficial across all conditions, its magnitude declines as perceived economic instability intensifies. This represents a dampening effect rather than a reversal. Institutional support continues to matter, yet macroeconomic uncertainty constrains the psychological translation of institutional learning into stronger ideational confidence.

This pattern contributes to ongoing debates concerning whether economic hardship stimulates or suppresses entrepreneurial behavior. ^13^ ^,^ ^36^ ^,^ ^39^ ^,^ ^68^ While crisis-driven entrepreneurship has been documented in informal sectors, the present findings suggest a more nuanced mechanism within higher education contexts. Economic instability may not eliminate ideation, but it attenuates the strength with which structured institutional engagement enhances opportunity confidence. Students may continue generating ideas, yet macroeconomic uncertainty tempers their perceived viability and implementation confidence.

The moderation effect demonstrates that microsystem-level institutional processes and exosystem-level economic perceptions operate interdependently in shaping entrepreneurial ideation. While prior entrepreneurship studies frequently invoke ecological systems theory as a descriptive framework to contextualise environmental influences, empirical examinations of cross-level moderation within this perspective remain limited. By statistically modelling the interaction between microsystem-level institutional engagement and exosystem-level economic perceptions, this study moves ecological theorising beyond conceptual application and provides quantitative evidence of interdependent environmental dynamics shaping entrepreneurial ideation.

The findings further resonate with institutional theory and perspectives on entrepreneurial agency. Institutional environments provide structured resources, legitimacy signals, and opportunity scaffolding. However, entrepreneurial agency is filtered through subjective interpretations of structural constraints. When students perceive the macroeconomic environment as volatile, their confidence in transforming ideas into viable ventures becomes attenuated, even when institutional support is available. Entrepreneurial ideation thus reflects the dynamic interplay between institutional scaffolding and structural interpretation.

Taken together, the findings indicate that entrepreneurship centre engagement exerts a strong developmental influence, while macroeconomic perceptions shape opportunity evaluation and condition the magnitude of ideational outcomes. Innovative business idea generation among university students therefore reflects an ecological interaction between institutional engagement and broader economic context.

## 7. Conclusion and implications

This study examined how entrepreneurship centre engagement influences innovative business idea generation among university students and how this relationship is conditioned by general economic conditions. Drawing on ecological systems theory, the study conceptualised entrepreneurial ideation as the outcome of interactions between institutional learning environments and broader structural forces.

The findings show that engagement with entrepreneurship centres strongly enhances innovative business idea generation. Students who actively participate in mentoring, training, and incubation activities demonstrate stronger ideational capacity. This confirms that entrepreneurship centres function as meaningful developmental environments within the educational microsystem.

General economic conditions also play a significant role. Students’ perceptions of macroeconomic stability positively influence idea generation, while adverse economic perceptions weaken the positive effect of entrepreneurship centre engagement. The moderation analysis reveals that institutional support remains beneficial across all contexts, yet its strength is attenuated when students perceive economic instability as severe.

By integrating microsystem and exosystem influences, this study extends the application of ecological systems theory to entrepreneurship education research. The findings show that institutional mechanisms cannot be evaluated independently of broader economic realities. Entrepreneurial outcomes emerge from the interaction between proximate educational environments and macroeconomic structures.

Although the model demonstrates moderate explanatory power and predictive relevance, the results are derived from a cross-sectional design within a single institutional context. The findings therefore, provide insight into associative relationships rather than causal claims and should be interpreted within the boundaries of the sampled environment.

The study contributes to entrepreneurship education scholarship by clarifying how university-based entrepreneurial ecosystems operate within emerging economy conditions characterised by structural uncertainty.

### 7.1 Theoretical implications

This study advances entrepreneurship research in several important ways.

First, it extends the explanatory power of Ecological Systems Theory by operationalising it as a mechanism-based multilevel framework rather than a descriptive backdrop. The findings empirically demonstrate that entrepreneurship centres function as microsystem structures that shape entrepreneurial cognition through repeated and structured interaction. At the same time, perceived general economic conditions operate within the exosystem by influencing opportunity evaluation and feasibility perception. The statistically significant interaction effect provides empirical confirmation that these systems operate interdependently. This moves ecological theorising in entrepreneurship beyond single-level applications and demonstrates how cross-level dynamics shape ideational outcomes.

Second, the study contributes to debates concerning the relationship between macroeconomic instability and entrepreneurial behaviour. Rather than endorsing a simplified necessity-driven entrepreneurship narrative, the findings reveal a dampening mechanism. This finding refines crisis-activation narratives by distinguishing between motivational activation and ideational strengthening and clarifies how structural instability shapes entrepreneurial cognition within educational settings.

Third, the study advances institutional entrepreneurship research by empirically demonstrating how structured university ecosystems influence entrepreneurial cognition. While prior research has emphasised institutional quality at the national level, this study shows that institutional scaffolding within universities plays a measurable developmental role. Entrepreneurship centres are not merely symbolic policy responses but function as proximal developmental environments that shape opportunity recognition and creative confidence when engagement is substantive.

Fourth, the research responds to growing calls for contextualised entrepreneurship education scholarship. ^2^ ^,^ ^69^ By modelling perceived macroeconomic conditions as a moderator, the study integrates structural context into institutional analysis. This approach moves beyond isolated programme evaluation and situates entrepreneurship education within broader ecosystemic realities, particularly in emerging economies where macroeconomic volatility is pronounced. Importantly, this positions entrepreneurial ideation as an ecological outcome shaped by nested institutional and structural forces, thereby advancing theoretical integration across entrepreneurship education, institutional theory, and macro-context research.

### 7.2 Practical and policy implications

The findings generate actionable implications for universities, policymakers, and ecosystem actors, particularly within emerging economy contexts.

For universities, the results demonstrate that the existence of an entrepreneurship centre alone is insufficient. The magnitude of ideational outcomes depends on sustained and meaningful engagement. Institutions should therefore prioritise structured mentoring pathways, incubation continuity, interdisciplinary collaboration, and iterative experiential learning. Programme design should emphasise depth of engagement rather than symbolic participation. Monitoring engagement intensity can serve as an internal performance indicator for entrepreneurship centres.

The moderation findings further suggest that entrepreneurship training must incorporate economic realism. Since perceived macroeconomic instability dampens ideational strengthening, universities should integrate modules on adaptive strategy, financial resilience, resource bootstrapping, and opportunity evaluation under uncertainty. Equipping students with tools to navigate volatile environments may mitigate the constraining influence of adverse economic perceptions.

For policymakers, the study highlights that entrepreneurship education policy cannot be decoupled from macroeconomic stability efforts. Institutional interventions at the university level are most effective when embedded within supportive economic environments. Policies aimed at improving regulatory quality, expanding access to finance, stabilising infrastructure, and reducing inflationary pressures indirectly enhance the developmental impact of entrepreneurship centres. Educational reform and macroeconomic reform should therefore be treated as complementary rather than independent policy domains.

The findings also underscore the importance of integrating university entrepreneurship centres into broader innovation systems. Stronger linkages with industry partners, financial institutions, development agencies, and regional innovation hubs can buffer students from macroeconomic uncertainty by expanding resource networks and opportunity visibility. Such ecosystem integration may attenuate the dampening moderation effect observed in the study.

For emerging economies more broadly, the results suggest that entrepreneurship education must be embedded within coordinated ecosystem strategies. Institutional strengthening without structural stability limits ideational potential. Sustainable entrepreneurial development therefore requires alignment between university-based support mechanisms and national economic governance structures.

Furthermore, the predictive relevance of the model indicates that measuring both engagement intensity and economic perception can serve as diagnostic tools for programme evaluation. Data-driven monitoring enables institutions to refine resource allocation, strengthen engagement mechanisms, and identify contextual vulnerabilities that may constrain entrepreneurial development.

### 7.3 Limitations and suggestions for further research

Several limitations should be considered when interpreting the findings. First, the study adopts a cross-sectional design. Although the hypothesised relationships are theoretically grounded and statistically supported, the design does not permit causal inference or examination of temporal dynamics. The associations reflect contemporaneous relationships rather than developmental trajectories. Future research should employ longitudinal or panel designs to assess whether sustained engagement with entrepreneurship centres produces cumulative ideational gains or whether the strength of these effects varies across changing economic cycles.

Second, the empirical context is limited to a single university in Nigeria with a relatively structured entrepreneurship ecosystem. While this setting provides a suitable environment for theory testing, institutional characteristics may shape the magnitude of observed relationships. Multi-institutional studies across universities with varying levels of entrepreneurship infrastructure would strengthen external validity. Cross-national research across emerging and developed economies would further clarify whether the moderating influence of economic perceptions operates consistently across distinct structural contexts.

Third, general economic conditions were measured perceptually. This approach is theoretically justified because entrepreneurial cognition is shaped by subjective interpretation of structural signals. However, future research could integrate objective macroeconomic indicators such as inflation rates, credit availability measures, regulatory quality indices, or regional business density statistics. Combining perceptual and archival economic data would allow examination of whether subjective and objective conditions exert differential or interactive moderating effects.

Fourth, the use of self-reported survey data introduces potential common method bias despite procedural and statistical controls. Future studies could incorporate multi-source designs, including institutional participation records, mentor assessments, behavioural innovation outputs, or venture creation data. Such approaches would strengthen construct validation and provide behavioural corroboration of ideational outcomes.

Fifth, although the explanatory power observed in this study is consistent with established benchmarks for behavioural research, some prior studies have reported higher predictive values when incorporating additional psychological and behavioural antecedents. Entrepreneurial ideation is inherently multidimensional and may also be influenced by unobserved individual-level factors such as entrepreneurial self-efficacy, intrinsic motivation, prior entrepreneurial exposure, personality traits, and social capital. The exclusion of such variables may limit the total variance explained, and future research integrating psychological, relational, and contextual determinants within a multilevel framework could yield stronger predictive precision and deeper theoretical insight.

Sixth, the moderation analysis identifies a linear dampening effect of perceived economic instability. However, it does not examine potential non-linear or threshold dynamics. Extremely adverse conditions may stimulate necessity-driven ideation rather than suppression. Future research could test curvilinear relationships, interaction thresholds, or subgroup differences to explore whether tipping points exist in the relationship between economic perception and ideational outcomes.

Furthermore, while Partial Least Squares Structural Equation Modelling was appropriate for predictive modelling and theory development, confirmatory covariance-based approaches may be employed in future studies as theoretical relationships mature. Qualitative inquiry could also enrich understanding by examining how students interpret macroeconomic uncertainty and how institutional engagement shapes opportunity framing in practice.

Despite these limitations, the study provides an integrated multilevel framework for understanding how university-based entrepreneurship ecosystems interact with broader economic structures. By identifying both institutional and contextual determinants of innovative business idea generation, the research establishes a foundation for longitudinal, cross-institutional, and cross-national investigations that can further refine theory and inform entrepreneurship policy in emerging economies.

## Consent to participate

All participants voluntarily consented to participate in the study after being informed about its purpose and their rights. No personally identifiable data was collected, and confidentiality was strictly maintained throughout.

## Author contributions

**Jane O. Ben-Caleb:** Conceptualization, Data Curation, Methodology, Formal Analysis, Software, Funding Acquisition, Investigation, Resources, Validation, Writing – Original Draft Preparation, Writing – Review & Editing.

**Itumeleng Ngowi:** Funding Acquisition, Investigation, Writing – Review & Editing.

**Adebanji A.W. Ayeni:** Data Curation, Funding Acquisition, Methodology, Project Administration, Validation, Visualization, Writing – Review & Editing.

**Ayandeji Sunday Ayantokun:** Funding Acquisition, Writing – Review & Editing.

**Henry E. Inegbedion:** Data Curation, Supervision, Writing – Review & Editing.

**Egbide Ben-Caleb:** Conceptualization, Supervision, Validation, Writing – Original Draft Preparation.

## Copyright

© 2025 Ben-Caleb et al. This work is licensed under a Creative Commons Attribution 4.0 International (CC BY 4.0) license.

## Data availability

The dataset and extended materials supporting the findings of this study are openly available on Zenodo at https://doi.org/10.5281/zenodo.16403311 ^70^ under a Creative Commons Attribution 4.0 International (CC BY 4.0) license. The upload includes the DATASHARE information file, the structured questionnaire and measurement items used during data collection (Appendix A), as well as supplementary materials comprising the measurement model output tables, including the Fornell-Larcker Criterion and cross-loadings. These materials are sufficient to reproduce the key analyses and support the transparency and replicability of the study. All files are freely accessible and may be reused with an appropriate citation.
