# Supplementary material for: Innovative Business Idea Generation in Higher Education: The Interplay between Entrepreneurship Centres and Economic Conditions
Source: F1000Res. 2026 Jul 27;14:867. Originally published 2025 Sep 3. [Version 2] doi: 10.12688/f1000research.167767.2 (PMC13562960; doi:10.12688/f1000research.167767.2)
Supplement: Peer review history

# Reviewer report: Reviewer response for version 2

**DOI:** 10.5256/f1000research.198153.r505298

**Published:** 2026-09-01

**Authors:** Abid Hossain Shawon

**Affiliations:** 1 Zhengzhou University, Zhengzhou, Henan, China

I am pleased to provide my final recommendation regarding the above-referenced manuscript, which I was invited to review.

 The authors have submitted a revised version of their manuscript, and I have carefully evaluated the changes made in response to my previous review comments. I am satisfied that the authors have addressed the concerns raised in the initial review in a thorough and constructive manner.

Is the work clearly and accurately presented and does it cite the current literature?

Yes

If applicable, is the statistical analysis and its interpretation appropriate?

Partly

Are all the source data underlying the results available to ensure full reproducibility?

Partly

Is the study design appropriate and is the work technically sound?

Partly

Are the conclusions drawn adequately supported by the results?

Yes

Are sufficient details of methods and analysis provided to allow replication by others?

Yes

Reviewer Expertise:

National Economics, Social Entrepreneurship, Poverty Alleviation, Corporate Governance, SDGs and ESGs

I confirm that I have read this submission and believe that I have an appropriate level of expertise to confirm that it is of an acceptable scientific standard.

# Reviewer report: Reviewer response for version 1

**DOI:** 10.5256/f1000research.184908.r461226

**Published:** 2026-03-09

**Authors:** Antonio Rodríguez Peña

**Affiliations:** 1 Ean University, Bogotá, Colombia

Thank you for the opportunity to review this manuscript. The paper tackles a relevant and timely question, and it offers useful empirical evidence on how university entrepreneurship centres may influence innovative idea generation, particularly in contexts shaped by economic constraints. The study is clearly structured, the empirical strategy is generally appropriate, and the results are presented in a transparent way.

 That said, there are several areas where the manuscript could be strengthened in order to sharpen its theoretical contribution.

 First, the theoretical framing would benefit from further development. While Ecological Systems Theory provides an interesting overarching lens, its explanatory role in the model remains somewhat implicit. It would be helpful to clarify more explicitly why this framework is the most suitable choice for the research question and to articulate the specific mechanisms through which the different “systems” influence idea generation. Bringing in complementary perspectives—such as institutional theory, entrepreneurial agency, or resilience—could also help deepen the interpretation of the findings.

 Second, the measurement of General Economic Conditions (GEC) deserves additional discussion. Measuring economic conditions through perceptions is defensible, but the manuscript would be stronger with a clearer conceptual justification for privileging subjective assessments over objective macroeconomic indicators. It would also be useful to reflect briefly on potential common method concerns associated with perceptual measures collected from a single survey.

 Third, although the PLS-SEM analysis is competently executed, the methodological section could be reinforced through additional robustness checks. For example, explicitly addressing common method bias, discussing possible endogeneity issues, and providing a graphical illustration of the moderating effect would increase confidence in the stability and interpretation of the results.

 Fourth, the discussion section could engage more deeply with entrepreneurship theory. The negative moderation effect of economic conditions is a particularly interesting result, and it would benefit from a richer theoretical conversation about how environmental constraints shape entrepreneurial agency, opportunity perception, and action. This would elevate the contribution beyond the immediate empirical setting.

 Finally, given the context-specific nature of the sample, a more explicit reflection on the limits of generalizability would strengthen the manuscript. Clarifying how the findings might (or might not) extend to other emerging economies would help situate the contribution more clearly.

 Overall, the manuscript rests on a solid empirical foundation and addresses a meaningful research question. With a more fully developed theoretical articulation and some additional methodological clarification, it has clear potential to make a stronger contribution to the literature on entrepreneurship and higher education.

Is the work clearly and accurately presented and does it cite the current literature?

Yes

If applicable, is the statistical analysis and its interpretation appropriate?

Partly

Are all the source data underlying the results available to ensure full reproducibility?

Yes

Is the study design appropriate and is the work technically sound?

Partly

Are the conclusions drawn adequately supported by the results?

Partly

Are sufficient details of methods and analysis provided to allow replication by others?

Partly

Reviewer Expertise:

Corporate entrepreneurship Sustainable Project Management Sustainable Development Goals PLS-SEM

I confirm that I have read this submission and believe that I have an appropriate level of expertise to confirm that it is of an acceptable scientific standard, however I have significant reservations, as outlined above.

# Reviewer report: Reviewer response for version 1

**DOI:** 10.5256/f1000research.184908.r461228

**Published:** 2026-02-27

**Authors:** Nurul Hidayana Mohd Noor

**Affiliations:** 1 Faculty of Administrative Science and Policy Studies, Universiti Teknologi MARA, Seremban, Negeri Sembilan, Malaysia

1. The authors do a good job explaining key statistics related to unemployment. However, the discussion would be strengthened by clearly adding a problem statement that highlights the link between unemployment and the importance of entrepreneurship. This addition would help frame the analysis more effectively and underscore why entrepreneurship is a critical response to the unemployment challenge.
2. Could you please explain or provide examples of some entrepreneurship centers in Nigerian universities and describe the activities they undertake? Including specific centers and what they do would help illustrate how entrepreneurship is being supported in the academic context.
3. The introduction lacks critical discussion of the problem statement. In addition, the authors fail to clearly identify the research gaps and adequately discuss the significance of the study.
4. The Introduction section primarily focuses on entrepreneurship centres. However, it does not introduce or discuss other key variables that are central to the study, such as Innovative Business Idea Generation and General Economic Conditions as a moderating variable. Including these elements in the introduction would provide a clearer context for the research framework and the rationale behind the study.
5. Additionally, it would strengthen the section if the authors could list the research objectives or research questions at the end of the introduction, providing readers with a clear understanding of the study’s focus and scope.
6. The manuscript lacks in-depth discussion of each proposed hypothesis. Most of the discussion is too descriptive and does not provide sufficient critical analysis or supporting evidence from prior studies. The authors are encouraged to expand the discussion, critically examining each hypothesis and providing relevant empirical or theoretical support to strengthen the manuscript.
7. The manuscript does not explain how the sample size was determined. The authors should provide a clear justification or calculation method for the sample size, including any assumptions, formulas, or references used, to ensure the study’s validity and reliability.
8. The manuscript requires further elaboration on the sampling technique and data collection process. The authors should clearly describe the sampling method used, inclusion/exclusion criteria, recruitment procedures, and data collection steps to ensure transparency and reproducibility of the study.
9. In the section on measuring the variables, the authors need to clearly state the sources of the measurement instruments, including references to prior validated scales or studies. This will help establish the reliability and validity of the measurements used in the study.
10. In the Discussion section, the manuscript fails to adequately support the findings with past studies. The authors are encouraged to integrate relevant literature to critically compare their results with previous research, which will strengthen the credibility and scholarly contribution of the study.
11. The manuscript lacks a discussion of practical implications. The authors should clearly explain how the findings can be applied in real-world contexts, such as in policy, entrepreneurship education, or business practice, to enhance the study’s relevance and impact.

Is the work clearly and accurately presented and does it cite the current literature?

Partly

If applicable, is the statistical analysis and its interpretation appropriate?

Yes

Are all the source data underlying the results available to ensure full reproducibility?

Yes

Is the study design appropriate and is the work technically sound?

Yes

Are the conclusions drawn adequately supported by the results?

Yes

Are sufficient details of methods and analysis provided to allow replication by others?

Yes

Reviewer Expertise:

entrepreneurship, policy studies, gender studies, and management

I confirm that I have read this submission and believe that I have an appropriate level of expertise to confirm that it is of an acceptable scientific standard, however I have significant reservations, as outlined above.

# Reviewer report: Reviewer response for version 1

**DOI:** 10.5256/f1000research.184908.r413971

**Published:** 2025-09-26

**Authors:** Abid Hossain Shawon

**Affiliations:** 1 Zhengzhou University, Zhengzhou, Henan, China

The topic is timely and important: university entrepreneurship centers and the boundary role of macroeconomic conditions in shaping student idea generation is of clear interest to educators, policy makers and entrepreneurship scholars. The paper contains promising empirical work and useful policy discussion. However, substantial weaknesses in methodological transparency, measurement reporting, theoretical integration, and presentation prevent acceptance in its current form. The authors should revise following the points below and submit a full, tracked-changes revision with a point-by-point response.

 1.Provide full details of the sampling frame (universities, selection criteria, why North-Central only), response rate, and inclusion criteria. Justify sample size (power analysis or SEM rules) and discuss potential sampling bias.

 2.Clarify the novelty of the study, sharpen research questions and hypotheses, and explain why Ecological Systems Theory is the right lens. Strengthen the literature review by identifying clear gaps and linking hypotheses to EST propositions. The following articles will enhance your literature review parts

*(refer to 1,2,3)*

 3. Report a full measurement model table (all loadings, α, CR, AVE, rho_A). Include Fornell–Larcker, cross-loadings, and exact item wording (appendix). Explain why indicators were removed and discuss implications.

 4.Justify use of student perceptions as a GEC measure. Provide validity evidence, source, and rationale. Consider alternative specifications (formative vs reflective) and note limitations vs objective macro indicators.

 5.Test for common method variance (Harman’s, VIF, marker variable) and discuss endogeneity concerns. Avoid causal claims from cross-sectional data.

 6.Report exact p-values, confidence intervals, effect sizes (f²), SRMR, and model fit indices. Revise Table 5 criteria (align with standard p < .05 unless justified otherwise). Provide robustness checks (alternative interaction methods, controls such as gender, prior experience).

 7.Plot and interpret the interaction (simple slopes at low/medium/high GEC). Discuss substantive meaning and nuanced policy implications.

 8.Limitations & future research: Expand to include self-report bias, single-region sample, and cross-sectional design. Suggest multi-university or longitudinal studies and objective GEC indicators.

Is the work clearly and accurately presented and does it cite the current literature?

Yes

If applicable, is the statistical analysis and its interpretation appropriate?

Partly

Are all the source data underlying the results available to ensure full reproducibility?

Partly

Is the study design appropriate and is the work technically sound?

Partly

Are the conclusions drawn adequately supported by the results?

Yes

Are sufficient details of methods and analysis provided to allow replication by others?

Yes

Reviewer Expertise:

National Economics, Social Entrepreneurship, Poverty Alleviation, Corporate Governance, SDGs and ESGs

I confirm that I have read this submission and believe that I have an appropriate level of expertise to confirm that it is of an acceptable scientific standard, however I have significant reservations, as outlined above.

## References

[1] : Analyzing the Contribution of Social Microfinance to Rural Financial Progress: Entrepreneurial and Social Dimensions in a Developing Nation. *Indus Journal of Social Sciences* .2025;3(1) : 10.59075/ijss.v3i1.717505-525 10.59075/ijss.v3i1.717

[2] : Analyzing the impact of financial development, human capital and industrial structure on ecological footprints: a PMG-ARDL approach. *Humanities and Social Sciences Communications* .2025;12(1) : 10.1057/s41599-025-05553-w 10.1057/s41599-025-05553-w

[3] : Assessing the Impact of Institutional Entrepreneurship, Social Innovation, and Social Enterprise on Poverty Alleviation: Insights From South Asian Economies. *Sustainable Development* .2025;33(4) : 10.1002/sd.34195528-5544 10.1002/sd.3419
